# uPAR exhibits age- and region-dependent expression in the brains of mice with Alzheimer’s disease-like pathology

**DOI:** 10.1016/j.brainres.2026.150364

**Published:** 2026-05-02

**Authors:** Lauren E. Sarko, Ella Klaus, Viktoriya Bondarenko, Callista Secker, Mackenzie S. Mnuk, Kaitlyn M. Marino, Daniel C. Shippy, Tyler K. Ulland, Krishanu Saha, Marina E. Emborg, Jeanette M. Metzger

**Affiliations:** a Cellular and Molecular Pathology Graduate Program, University of Wisconsin–Madison, Madison, WI, USA; b Wisconsin Institute for Discovery, University of Wisconsin–Madison, Madison, WI, USA; c Wisconsin National Primate Research Center, University of Wisconsin–Madison, Madison, WI, USA; d Department of Pathology and Laboratory Medicine, University of Wisconsin–Madison, Madison, WI, USA; e Wisconsin Alzheimer’s Disease Research Center, University of Wisconsin–Madison, Madison, WI, USA; f Department of Biomedical Engineering, University of Wisconsin–Madison, Madison, WI, USA; g Department of Medical Physics, University of Wisconsin–Madison, Madison, WI, USA

**Keywords:** Alzheimer’sDisease (AD), Senescence, Neurodegeneration, uPAR, 5xFAD, Mouse, Brain

## Abstract

The urokinase-type plasminogen activator receptor (uPAR) is a GPI-anchored cell surface protein that regulates leukocyte adhesion, migration, and activation, thereby contributing to inflammation and tissue remodeling. However, its role in Alzheimer’s disease (AD), particularly in relation to glial dysfunction, remains poorly defined. Here, we investigated the temporal and spatial regulation of uPAR expression across AD mouse models with intact or deficient adaptive immunity. Using immunohistochemistry, we assessed uPAR expression in Rag2/Il2rg^−/−^ (Rag), Rag2/Il2rg^−/−^-5xFAD (Rag-5xFAD), C57BL/6 (WT), and 5xFAD mice across multiple brain regions. uPAR expression increased with age and was significantly elevated in 5xFAD mice, with robust upregulation evident by 6 months irrespective of immune status. Immunofluorescence revealed that uPAR localized predominantly to Iba1^+^ microglia clustered around Aβ plaques, with limited neuronal expression. Bulk RNA sequencing of Rag-5xFAD brain tissue demonstrated enrichment of disease-associated microglia (DAM) and senescence-related transcriptional programs. These findings indicate that uPAR marks a subset of plaque-associated glial cells undergoing functional and transcriptional remodeling in AD, independent of peripheral adaptive immune signaling. Collectively, our results identify uPAR as a marker of dysfunctional, DAM-like microglia and implicate it in senescence-associated neuroinflammatory pathways. This work provides a framework for future studies targeting uPAR-expressing glial populations as a potential therapeutic strategy in AD.

## 1. Introduction

Alzheimer’s disease (AD) is the leading cause of age-related pathological neurodegeneration, affecting an estimated 7.2 million people in the United States alone (Alzheimer’s Association, 2025). The key neuropathological features, extracellular amyloid-β (Aβ) plaques and intracellular neurofibrillary tangles (NFTs), alone do not fully account for the complex mechanisms underlying disease progression. Microglia initially respond to Aβ deposits by attempting to phagocytose plaques via receptors such as TREM2 and CD36 (Lee and Landreth, 2010). However, chronic stimulation by persistent amyloid deposition can drive microglia toward a pro-inflammatory, disease-associated state characterized by NF-κB activation and secretion of cytokines including IL-1β, IL-6, and TNF-α (Heneka et al., 2015; Keren-Shaul et al., 2017; Marino et al., 2023; Martínez-Cué and Rueda, 2020; Zhang et al., 2026). This chronic inflammatory signaling impairs microglial homeostasis, reduces phagocytic capacity, and establishes a self-perpetuating cycle of reactive microgliosis that promotes senescence-like phenotypes (Doens and Fernández, 2014).

Cellular senescence is a multifaceted stress response to endogenous and exogenous signals that contributes to chronic inflammation and tissue dysfunction (Baker and Petersen, 2018; Campisi and d’Adda di Fagagna, 2007; Muñoz-Espín and Serrano, 2014). A fundamental feature of senescence is the senescence-associated secretory phenotype (SASP), characterized by the secretion of tissue-specific inflammatory, oxidative, and matrix-degrading factors (Birch and Gil, 2020; Coppé et al., 2010; van Deursen, 2014; Walton et al., 2020). In the brain, senescence has been observed in multiple cell types, including microglia and astrocytes, with observed increased expression of p16^INK4A^, p21^WAF1/CIP1^, and SASP genes in age-related and neurodegenerative diseases such as AD (Alshaebi et al., 2025). These findings support a growing view that senescent glial cells contribute to AD pathology by establishing a chronic inflammatory environment promoting neurodegeneration. As a result, targeting glial senescence represents an emerging strategy to disrupt the chronic inflammatory milieu that drives disease progression (Lau et al., 2023; Riessland et al., 2024; Rim et al., 2024).

The urokinase-type plasminogen activator receptor (uPAR), also known as CD87, is a multifaceted cell surface receptor involved in diverse pathophysiological processes in both the central nervous system and the peripheral immune system. Although uPAR has been described in the context of aging and senescence-associated phenotypes, it is not a defining marker of cellular senescence (Amor et al., 2024; Byrns et al., 2024; Childs et al., 2015; Di Micco et al., 2021; Muñoz-Espín and Serrano, 2014). Rather, its expression is dynamically regulated and can be induced by a variety of stimuli, including tissue damage, neuroinflammation, and neuronal activity. In the brain, uPAR has been implicated in neuronal differentiation (Farias-Eisner et al., 2000), tissue repair (Merino et al., 2017), as well as microglial activation in response to inflammation (C. Cunningham et al., 2009; Liu, 2022; Liu and Chen, 2024; Mehra et al., 2016). Beyond the central nervous system, uPAR plays a role in immune cell migration, adhesion, and proteolytic signaling, highlighting its importance in coordinating immune responses under both physiological and pathological conditions (Iftikhar et al., 2025; Rasmussen et al., 2021).

These observations suggest that increased uPAR expression in the brain reflects context-dependent cellular activation rather than a single pathological state. In the context of neurodegeneration, *PLAUR*, the gene that encodes for uPAR, has been reported to be upregulated in AD brain tissue, with high plasma uPAR levels associated with higher risk of AD (Liu and Chen, 2024). However, genetic studies examining *PLAUR* polymorphisms have not identified a significant association with AD susceptibility (Cetinsoy et al., 2024), suggesting that altered uPAR expression in AD is possibly driven by disease-associated environmental changes. Despite these observations, the role of uPAR in neurodegeneration and AD pathogenesis remains poorly defined.

To model AD-like Aβ pathology, 5xFAD mice have been engineered to overexpress human amyloid precursor protein (APP) and presenilin-1 (PSEN1) with five familial AD mutations (Oakley et al., 2006). These mutations result in robust amyloid pathology, with plaques appearing as early as 2–4 months old and continuing to accumulate with progressing age (Forner et al., 2021). The 5xFAD model exhibits strong microglial activation in parallel with rapid plaque accumulation, making it widely used to evaluate mechanisms of microglial dysfunction and test therapeutic strategies that target senescent or dysregulated glial cells (Oakley et al., 2006; Spangenberg et al., 2016). Rag-5xFAD immunodeficient mice generated by crossbreeding 5xFAD and Rag (Rag2/Il2rg double knockout) mice, which lack mature T, B, and NK cells (Marsh et al., 2016), provide a platform for examining how the lack of an adaptive immune system influences plaque pathology and microglial phenotypes. Previous investigation of Rag-5xFAD mice showed exacerbated Aβ-plaque pathology, altered microglia phenotype, and microglial-enriched genes relative to immunocompetent 5xFAD mice (Marsh et al., 2016).

Although many studies have investigated the expression of Aβ pathology in 5xFAD mice (Bader et al., 2023; Forner et al., 2021; Oblak et al., 2021; Oh et al., 2018), uPAR gene and protein expression have not been evaluated beyond an association with synaptic dysfunction (Diaz et al., 2020). To address this gap, here we report the quantification of uPAR protein expression in the primary motor cortex (M1), striatum, CA1, dentate gyrus (DG), subiculum, and thalamus of WT, Rag, 5xFAD, and Rag-5xFAD mice at 2, 4, and 6 months of age. We also evaluated gene expression of Rag-5xFAD vs. Rag mice to identify transcriptional differences in senescent- and disease-associated microglia genes. Overall, this study aimed to define the spatial and temporal patterns of uPAR expression in models of AD pathology and determine how these changes relate to disease-associated microglial states.

## 2. Materials and methods

### 2.1. Subjects

All animal experiments were approved by the University of Wisconsin-Madison Animal Care and Use Committee under protocols #M005915 and #M00609. Mice underwent a 12-hour light/dark cycle and were housed in a specific pathogen-free vivarium with strictly regulated temperature, pressure, and humidity controls to ensure a healthy, stable environment. Brain tissues from a total of 71 mice were analyzed in this study (Supp Table 1).

Tissues from immunocompetent and immunocompromised mice were utilized in this study. Immunocompetent mice were wild type (WT) C57BL/6J mice and hemizygous 5xFAD mice (Stock # 34848-JAX; Tg (APP,PSEN1)5xFAD+/−) with the same C57BL/6J background. The 5xFAD mice co-express human amyloid precursor protein (APP) and presenilin-1 (PSEN1) transgenes under the Thy1 promoter with five AD-linked mutations: APP K670_M671 delinsNL, APP I716V, APP V717I, PSEN1 M146L, and PSEN1 L286V (B6.Cg-Tg(APPSwFlLon, PSEN1*M146L*L286V)6799Vas/Mmjax).

Immunodeficient mice were Rag (Rag2^−/−^; Il2rg^−/−^ double knockout mice (Taconic, Model #4111-M; C57BL/6NTac.Cg-*Rag2*^*tm1Fwa*^ *Il2rg*^*tm1Wjl*^)) and Rag-5xFAD mice (Rag2^−/−^; Il2rg^−/−^; Tg(APP,PSEN1) 5xFAD^+/−^). Rag mice lack mature T and B lymphocytes due to Rag2 deficiency, lack NK cells due to Il2rg deficiency, and show loss of functional receptors for several cytokines (e.g.: IL-2, IL-4, IL-7, IL-9, and IL-15.); they are maintained on a C57BL/6NTac background. Rag-5xFAD mice were generated by crossing female 5xFAD^+/−^ mice with male Rag2^−/−^; Il2rg^−/−^ mice. Offspring were genotyped by PCR, and mice carrying both the double knockout alleles and the 5xFAD transgene were selected for study. A detailed description of Rag-5xFAD mice generated by crossing 5xFAD mice (Stock # 34848-JAX with a C57BL/6J background) and Rag mice (Taconic, Model #4111 with a C57BL/6NTac background) has been published elsewhere (Marsh et al., 2016).

### 2.2. Necropsy and tissue processing

Brain tissues were collected from two different sources. Wild type (WT) and 5xFAD mice were obtained from a previously published study (Marino et al., 2025). Rag and Rag-5xFAD mice were sourced and bred by the Biomedical Research Model Services (BRMS) and were processed similarly. Briefly, mice were anesthetized with isoflurane or CO_2_ and perfused with heparinized PBS. Brains were quickly harvested and either left whole or bisected. Whole brains and one of the brain hemispheres collected were postfixed in 4% paraformaldehyde (PFA) for 48 h. The remaining half brains were flash frozen in liquid nitrogen and stored at −80°C for RNA extraction (see additional methods below). The postfixed tissues were then rinsed, placed in graded sucrose solutions up to 30% for at least 48 h, frozen in a 2:1 30% sucrose/Tissue Tek OCT compound (Sakura #4583) solution, and cut into 40 μm coronal sections on a sliding microtome. The brain sections were stored at −20°C in cryoprotectant solution (1,000 mL × 1 PBS (pH 7.4), 600 g sucrose, 600 mL ethylene glycol) until immunostaining.

### 2.3. uPAR immunohistochemistry

Immunostaining for uPAR was performed following previously published protocols (Metzger et. al., 2023). Briefly, endogenous peroxidase activity was removed by a 20-minute incubation in 0.1 M sodium periodate. Non-specific staining was blocked using 5% normal serum in a tris-buffered saline solution containing 2% bovine serum albumin and 0.05% Triton X-100. The sections were incubated overnight in primary antibody (uPAR, 1:500, R&D Systems AF534, lot DCL0521042), washed in dilution media containing diH_2_O, NaCl, Tris-HCl, and Triton X-100, and incubated in secondary antibody (horse anti-goat, BA-9500, Vector Lab; 1:200) for one hour. Following a series of washes in dilution media, sections were incubated in the avidin biotin substrate (1:1000; ABC; Vector Laboratories) for 75 min. The sections were subsequently washed in 0.1 M imidazole/1.0 M acetate buffer (pH 7.4). Stains were visualized using 0.05% 3,3′-diaminobenzidine (DAB) and 0.05% H_2_O_2_. Tissue was mounted onto slides, counterstained with Nissl, allowed to dry, and cover slipped with EcoMount (EM897L, Biocare Medical). Negative and positive controls were processed in parallel for all immunostainings. Negative controls consisted of omitting the primary antibodies during the immunostaining. Positive controls were brain tissue sections from a high uPAR-expressing mouse not included in the main study, which was included in each immunostain to control for development time.

### 2.4. uPAR image acquisition and analysis

uPAR expression was evaluated in six brain regions of interest (ROIs): primary motor cortex (M1), striatum, CA1, dentate gyrus (DG), subiculum, and thalamus. The ROIs were identified in coronal tissue slices at the levels of the hippocampus and crossing of the anterior commissure (Allen Mouse Brain Coronal Atlas slides 79–85 and 47–56). Brightfield imaging was performed on a Nikon Eclipse Ti2 microscope with a DS-Ri2 camera and NIS Elements software. Stitched x/y images containing the entire ROI were collected with 30% overlap using the 10x objective. To aid in identification of ROI anatomical boundaries, stitched x/y images of the entire tissue section were also collected with 30% overlap using the 4x objective. Camera settings were kept consistent between images captured at the same magnification.

The percent area above threshold (%AAT) of uPAR-immunoreactivity (–ir), defined as the percentage of the pixels in the ROI that have a mean grey value above a predetermined threshold, was quantified in each of the six ROIs using FIJI software (Schindelin et al., 2012). Images were processed via background subtraction with rolling ball radius set to 300 pixels and the light background option. Brown uPAR-ir was separated from Nissl counterstaining using the ‘Colour Deconvolution’ plugin with the ‘H DAB’ option.

ROIs were drawn in original RGB photomicrographs for easier visualization of anatomy and then copied onto color deconvoluted images for analysis. For ROIs other than the thalamus, the entire anatomical area was outlined using the FIJI ‘Freehand selections’ tool with reference to the Allen Adult Mouse Coronal Brain Atlas to define ROI anatomy. Thalamic ROIs were drawn using the FIJI ‘Rectangle’ tool to draw a 0.4 mm square placed in the dorsal lateral edge of the thalamus. Any holes/tissue damage ≥ 0.003 mm^2^ within ROIs were excluded. The threshold used for calculating %AAT was determined per image to include all cellular, brown uPAR-ir and exclude any diffuse brown background, if present. Thresholds were set following consensus of two investigators across all images, with both investigators blind to the age, genotype, and sex of the animals.

### 2.5. Immunofluorescence

Fluorescence immunolabeling was performed to identify uPAR-expressing cell types and detect the presence of Aβ accumulation in the same subjects utilized for uPAR characterization, following previously validated methods (Metzger et al., 2023).

Brain tissue sections from selected animals of each experimental group underwent triple immunofluorescence labeling with antibodies against uPAR (AF534, R&D System; 1:500), the neuronal marker NeuN (ab279297, Abcam; 1:500), and the microglial marker ionized calcium binding adapter protein 1 (Iba1; 019–19741, WAKO,1:100). Briefly, the tissue sections were washed 3x for 20-minute in tris buffered saline (TBS) plus 0.05% TritonX-100, and background staining was blocked with a 2-hour incubation in a (TBS) solution containing 5% normal serum, 2% bovine serum albumin, and 0.05% Triton X-100. Tissue sections were then incubated with primary antibodies for 24 h at room temperature, washed 3x 20 min in dilution media, and then incubated for 2 h at room temperature with species appropriate Alexa Fluor 488, 594, and 647 secondary antibodies. After 3x 20 min dilution media washes, the tissue was counterstained with methoxy (Methoxy-XO4, Tocris 4920; 1:1000) to label Aβ accumulation, mounted onto slides, allowed to dry, and coverslipped with Fluor Gel. Each immunostaining included a negative control performed by omitting primary antibodies.

Additionally, brain tissue sections from 2- and 6-month old Rag-5xFAD animals were triple labeled against uPAR (AF534, R&D System; 1:500), NeuN (ab279297, Abcam; 1:500), and the astrocyte marker glial fibrillary acidic protein (GFAP; 13–0300, Invitrogen) and counterstained with 4′,6-diamidino-2-phenylindole (DAPI). The methods were the same as above, except that the uPAR primary and secondary were applied first, separately from the NeuN and GFAP primary and secondary antibodies, to avoid cross-reaction between the uPAR and GFAP primary antibodies.

Immunofluorescence imaging was performed using a Nikon A1R confocal microscope with 405, 488, 561, and 640 wavelength lasers using NIS Elements version 5.20.02. Detectors for the 488 and 561 lasers are high sensitivity GaAsP PMTs, while the 405 and 640 lasers use HS PMTs. Images were collected as 3x3 stitched 20x objective large images or as 2x2 stitched 40x objective large images merged over 3 Z-levels separated by 3 μm each.

### 2.6. RNA-sequencing

Snap-frozen Rag-5xFAD and Rag single-hemisphere brain tissue (Supp Table 1B) was homogenized on dry ice, and total RNA was extracted using an RNAeasy Mini Kit (Qiagen, Cat. No. 74104) with a purification of DNase with RNase-Free DNase Set (Qiagen, Cat. No. 79254). Quality and quantity of RNA was assessed using a N60 Nano-Photometer (IMPLEN). RNA library preparation and transcriptome sequencing were performed by Novogene using the Illumina NovaSeq 6000 Sequencing System. The libraries were built from 4 different mice per genotype (Rag-5xFAD and Rag) across 2 different timepoints (2 and 6 months). Sequences were aligned to the GRCm38/mm10 mouse genome.

### 2.7. Statistical analysis

Statistical analysis of uPAR-ir was performed in IBM SPSS Statistics v30. A 4 way (Age*Genotype*Sex*Brain ROI) repeated measures analysis of variance (ANOVA) was performed with sex, genotype, and age as between-subjects factors and brain ROI as the repeated measures, within-subjects factor (full results in Supp Table 2). As Mauchly’s test of sphericity indicated that the assumptions of sphericity had not been met (χ^2^(14) = 121.82, *p* = <0.001), Greenhouse-Geisser corrected p values are reported where applicable. Effect size for within- and between-subject effects are reported as partial eta squared (η^2^p). Results of pairwise comparisons are adjusted for multiple comparisons using the Bonferroni correction. A p < 0.05 was accepted as significant.

DEG (Differentially Expressed Genes) between Rag-5xFAD and Rag mice were determined by DESeq2 (Love et al., 2014) with log_2_FC > 1 and p < 0.05 considered statistically significant (Supp Tables 3 and 4). Benjamini-Hochberg procedure was used to adjust the p-values of individual genes. The volcano plots of DEGs were visualized by the ggplot R package in Rstudio. GSEA was analyzed using the clusterProfiler R package in RStudio. Heatmaps of DEGs and GSEA ranked genes were visualized by the ComplexHeatmap R package in Rstudio. GOBiological Process enrichment of DEGs were analyzed and visualized using the GOplot R package in Rstudio.

## 3. Results

### 3.1. uPAR expression was influenced by age, brain region, and immune condition

**Fig. 1.**
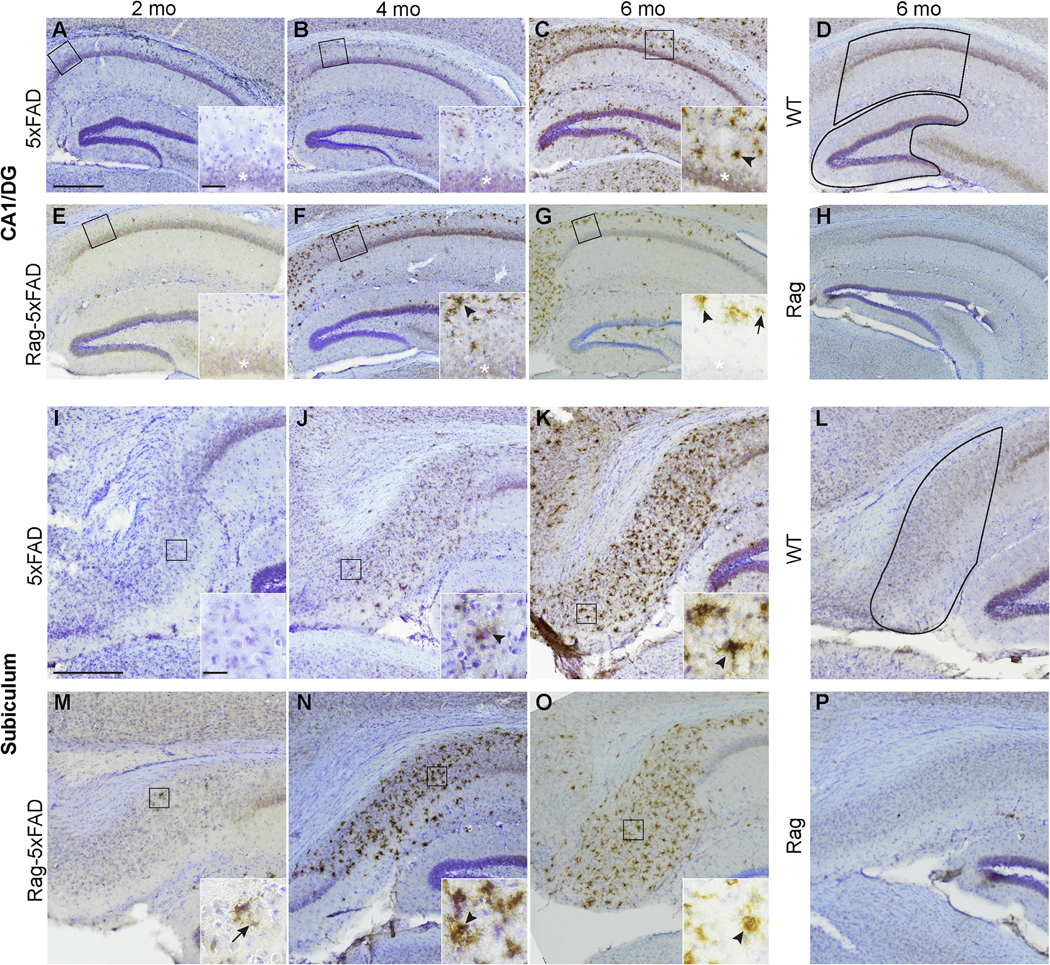
The hippocampal formation, particularly the subiculum, showed a dramatic increase in uPAR-immunoreactivity (–ir) with age in 5xFAD and Rag-5xFAD mice. Representative photomicrographs showing uPAR-ir (brown) in Nissl (blue) counterstained coronal mouse brain sections from wild type (WT; D, L), Rag (H, P), 5xFAD (A-C; I-K), and Rag-5xFAD (E-G; M–O) mice. Representative drawings of regions of interest (ROIs) used for quantification of uPAR-ir are shown in WT and Rag animals for CA1 and dentate gyrus (DG) in panel D and for the subiculum in panel L. Panel insets show high magnification images of representative uPAR-ir in each image, with black arrow heads indicating uPAR-ir ‘activated/ameboid’ microglia, while black arrows indicate less frequently observed uPAR-ir ‘ramified’ microglia. White asterisk identifies the location of CA1 pyramidal cell neuron bodies, which were often faintly uPAR-ir. Scale bars: 200 μm (main panels), 30 μm (panel insets).

**Fig. 2.**
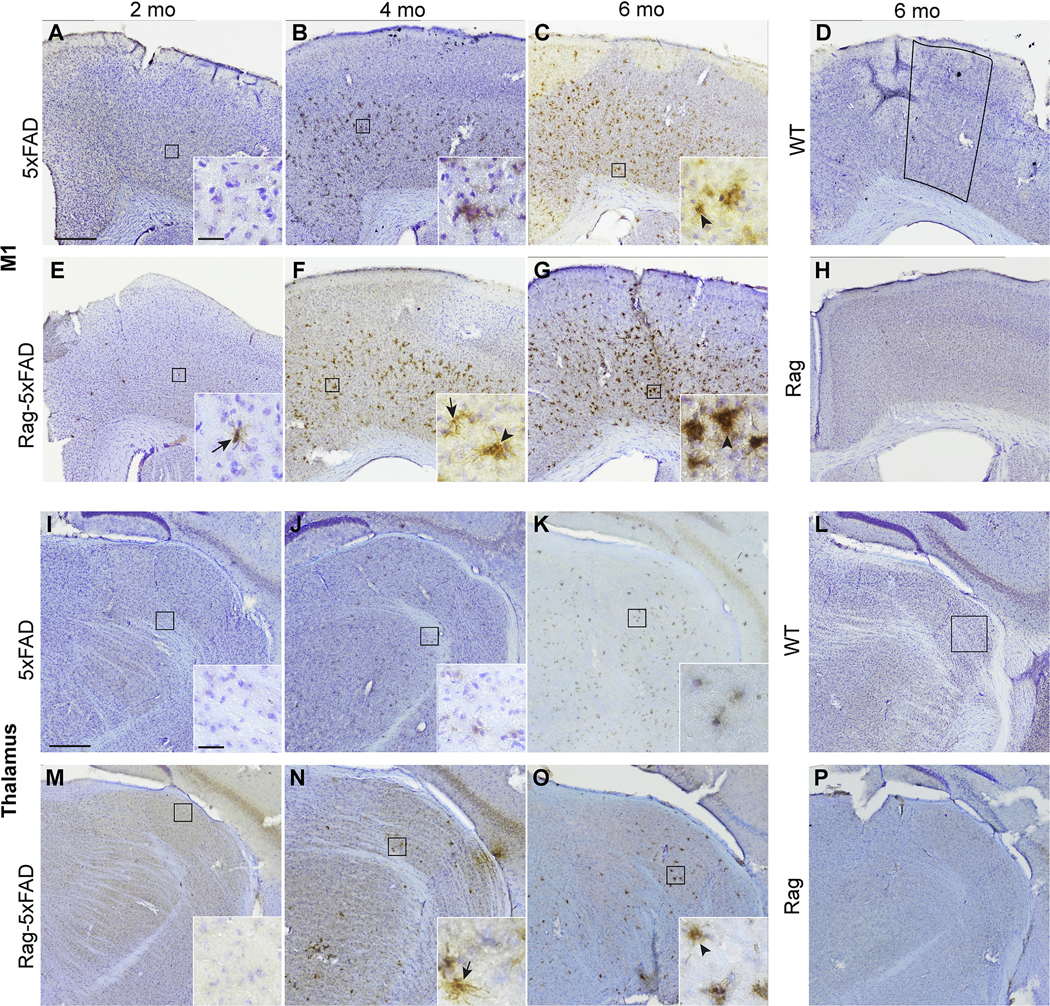
M1, and to a lesser extent the thalamus, showed an increase in uPAR-immunoreactivity (–ir) with age in 5xFAD and Rag-5xFAD mice. Representative photomicrographs showing uPAR-ir (brown) in Nissl (blue) counterstained coronal mouse brain sections from wild type (WT; D, L), Rag (H, P), 5xFAD (A-C; I-K), and Rag-5xFAD (E-G; M− O) mice. Representative drawings of regions of interest (ROIs) used for quantification of uPAR-ir are shown for M1 in panel D and for the thalamus in panel L. Panel insets show high magnification images of representative uPAR-ir in each image, with black arrow heads indicating uPAR-ir ‘activated/ameboid’ microglia. In contrast, black arrows indicate less frequently observed uPAR-ir ‘ramified’ microglia. Scale bars: 200 μm (main panels), 30 μm (panel insets).

**Fig. 3.**
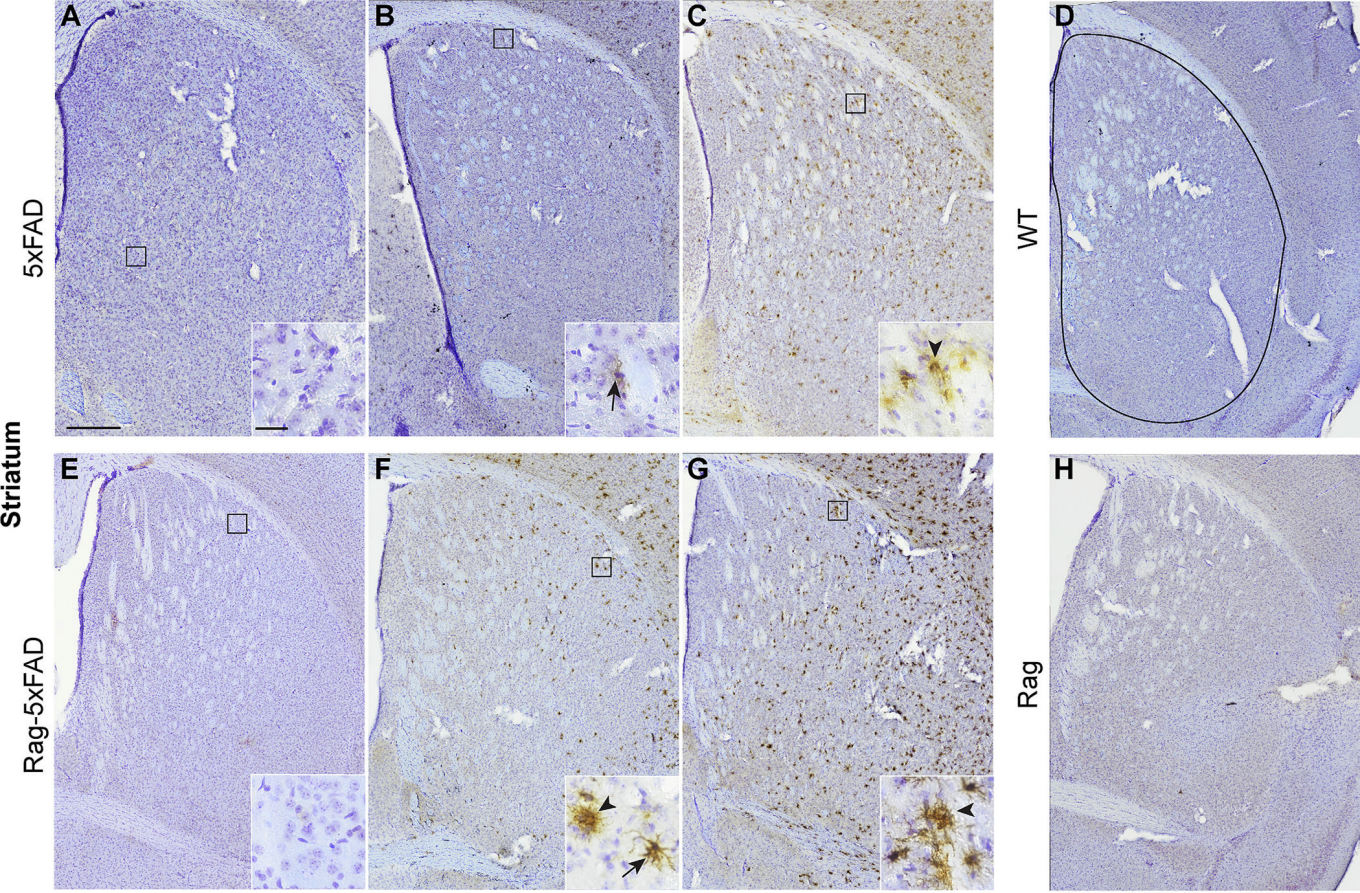
The striatum showed a moderate increase in uPAR-immunoreactivity (–ir) with age in 5xFAD and Rag-5xFAD mice. Representative photomicrographs showing uPAR-ir (brown) in Nissl (blue) counterstained coronal mouse brain sections from wild type (WT; A), Rag (H), 5xFAD (A-C), and Rag-5xFAD (E-G) mice. A representative drawing of the region of interest (ROI) used for quantification of uPAR-ir is shown in panel D. Panel insets show high magnification images of representative uPAR-ir in each image, with black arrow heads indicating uPAR-ir ‘activated/ameboid’ microglia. In contrast, black arrows indicate less frequently observed uPAR-ir ‘ramified’ microglia. Scale bars: 200 μm (main panels), 30 μm (panel insets).

Across all mouse ages (2, 4, and 6 months) and genotypes (WT, 5xFAD, Rag, and Rag-5xFAD), the hippocampal formation, including CA1 subfield, dentate gyrus (DG), and subiculum, as well as the thalamus, the primary motor cortex (M1), and the striatum were all recognizable and appeared anatomically normal. uPAR-ir was present in each of these brain regions, with considerable variability in cell density and expression intensity across ages and genotypes (Figs. 1–3). Several cell types expressed uPAR. Particularly intense uPAR-ir was observed in activated microglia-like cells exhibiting a rounded, ameboid shape, and uPAR-ir was also seen in cells with more ramified microglia-like morphology (Figs. 1–3 insets). Faint neuronal uPAR expression was mainly observed in the hippocampus and dentate gyrus (Fig. 1), and occasionally in other neuronal populations, such as M1 cortical neurons (Fig. 2).

**Fig. 4.**
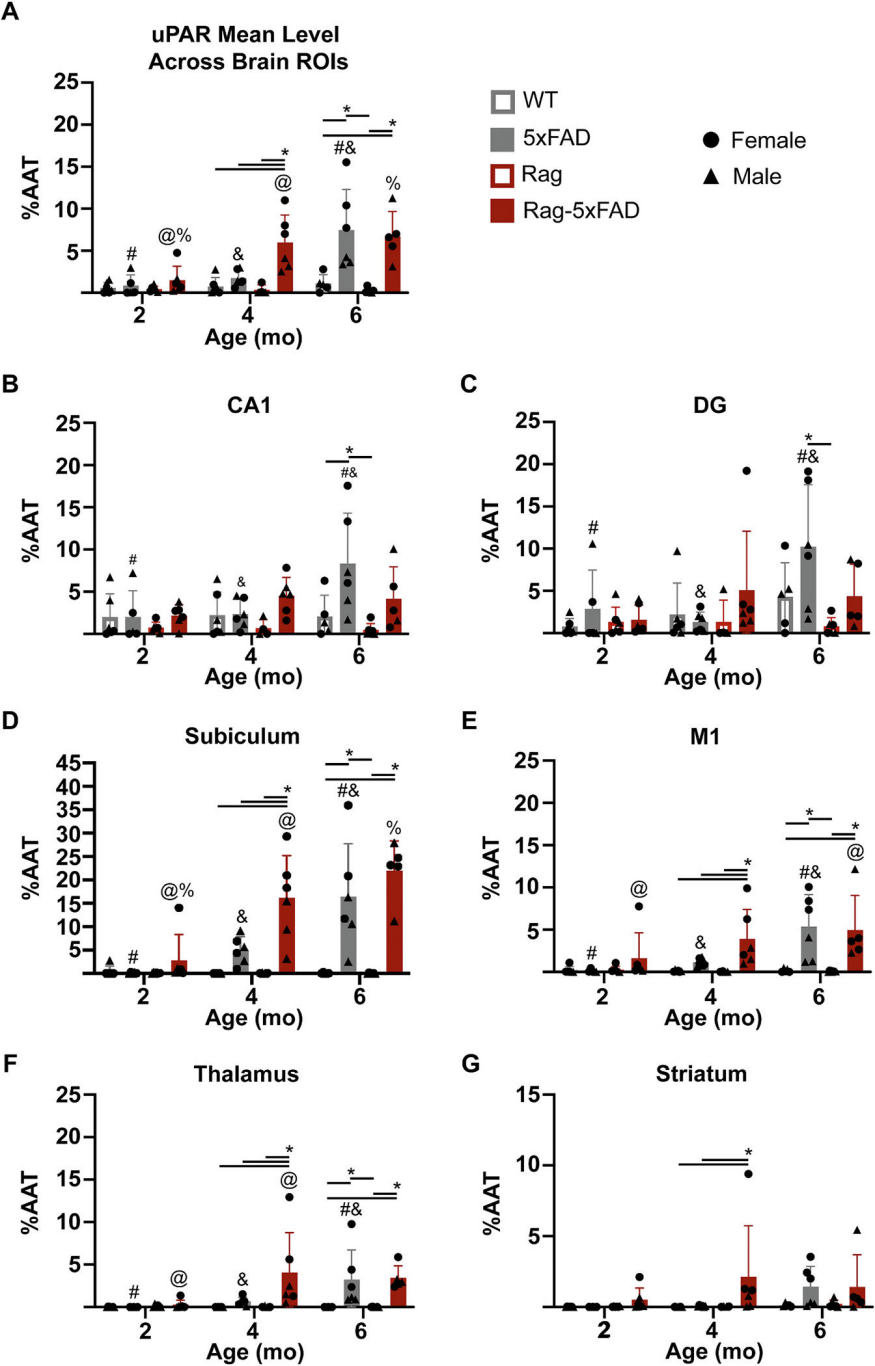
uPAR-immunoreactivity(-ir) in the brain significantly increased by 4 months of age in Rag-5xFAD mice and 6 months of age in 5xFAD mice. Graphs of uPAR-ir percent area above threshold (%AAT) data in all analyzed brain regions (A; mean of all brain regions), CA1 region of the hippocampus (B), dentate gyrus (DG; C), subiculum (D), M1 (E), thalamus (F), and striatum (G) of 2, 4, or 6 month old wild type (WT), Rag, 5xFAD, or Rag-5xFAD mice. Error bars show SD. Triangles represent individual %AAT values for male subjects, while circles represent female subjects. Lines in any graphs represent significant differences between two genotypes of the same age group (*, p < 0.05). Matching symbols (i.e., two of any symbol: #,&,@,%) represent a significant difference (p < 0.05) between two age groups of the same genotype. Detailed statistical results are provided in Supplementary Table 2.

Expression of uPAR was noticeably higher in older 5xFAD and Rag-5xFAD mice compared to younger, WT, or Rag animals (Figs. 1–4; Supp Table 2). Quantification of uPAR-ir confirmed that the presence and intensity of uPAR protein expression in the brain was significantly affected by animal age (p = 5.765E-6, η^2^p = 0.486), genotype (p = 2.680E-10, η^2^p = 0.665), and sex (p = 0.021, η^2^p = 0.117) and varied across brain ROIs (p = 7.299E-13, η^2^p = 0.412) (Fig. 4). At two months of age, uPAR-ir was not significantly different across genotypes. Notably, Rag-5xFAD animals appeared to accumulate uPAR-ir earlier than 5xFAD animals, as demonstrated by a significant increase in uPAR-ir at 4 months of age in Rag-5xFAD animals (Rag-5xFAD 2 vs. 4 month: p = 4.329E-5), which was not seen until 6 months of age in the 5xFAD group (5xFAD 2 vs. 6 month: p = 9.866E-8). This finding of accelerated timing of uPAR-ir accumulation in Rag-5xFAD mice compared to 5xFAD mice was further supported by a statistically significant difference between these two genotypes at 4 months (p = 2.102E-4); there was no significant difference between these groups at 6 months.

In both 5xFAD and Rag-5xFAD animals, uPAR-ir was particularly intense and accumulated earliest in the subiculum. In 4-month old Rag-5xFAD mice or 6-month old 5xFAD mice, the subiculum had significantly more uPAR-ir than almost any other region (4-month old Rag-5xFAD subiculum vs. other regions: CA1, p = 3.268E-6; DG, p = 3.886E-4; M1, p = 8.026E-8; striatum, p = 1.523E-8; thalamus, p = 1.012E-7 and 6-month old 5xFAD subiculum vs. other regions: CA1, p = 1.77E-3; M1, p = 1.012E-6; striatum, p = 3.097E-9; thalamus, p = 1.414E-8). Although containing less uPAR-ir than the subiculum, M1 showed moderately high uPAR-ir compared to other regions, with significantly more uPAR-ir compared to the striatum in 4-month (p = 7.374E-3) and 6-month (p = 2.677E-4) Rag-5xFAD mice and in 6-month 5xFAD mice (p = 2.946E-9). Interestingly, CA1 and DG neurons showed variably present uPAR-ir across animals in all groups (Fig. 1; Supp Fig. 1), consistent with somewhat higher and more variable uPAR-ir % AAT values in CA1 and DG of younger and WT or Rag animals compared to other brain regions (Fig. 4).

Female mice had higher levels of uPAR-ir than male mice at 4 months of age in Rag-5xFAD animals (p = 1.465E-4) and at 6 months of age in 5xFAD animals (p = 7.905E-7) when averaging over all brain regions. Moreover, this sex difference in uPAR-ir appears to have contributed to the observation that Rag-5xFAD animals demonstrated earlier increases in uPAR-ir compared to the immunocompetent 5xFAD. Female Rag-5xFAD animals showed a significant increase in uPAR-ir across regions from 2 to 4 months (2 vs. 4 month: p = 2.983E-5) and 6 months (2 vs. 6 month: p = 6.692E-3), while male Rag-5xFAD mice did not show a significant increase of uPAR until 6 months (4 vs. 6 month: p = 0.284). A similar trend was observed in 5xFAD mice, but with a slower time course: female mice had significant differences in uPAR levels between 4 and 6 months (p = 8.933E-9), while male mice lack significant difference between any timepoints.

### 3.2. uPAR was mainly expressed in lba1-ir microglia/macrophages

**Fig. 5.**
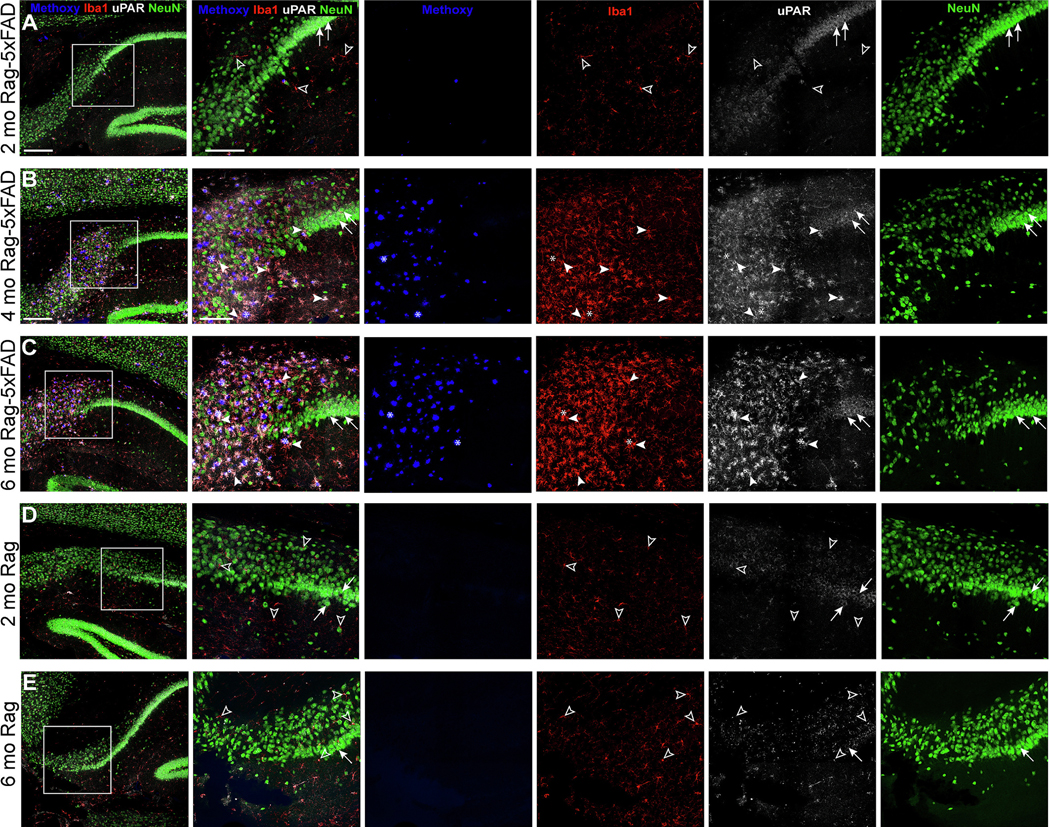
Expression of uPAR in Iba1-immunoreactive (–ir) microglia dramatically increased over time in Rag-5xFAD animals, but not in Rag animals. Representative photomicrographs of triple label immunofluorescence for the microglial marker Iba1 (red), uPAR (white), and the neuronal marker NeuN (green), counterstained with the fluorescent amyloid-beta (Aβ) probe methoxy-X04 (blue) in 2- (A), 4- (B), and 6-month (C) Rag-5xFAD mice and in 2- (D) and 6-month (E) Rag mice. A white box in the first column panel indicates the area of higher magnification shown in panels to the right for each row. Microglia immunoreactive for both Iba1 and uPAR appear pink (red Iba1 + white uPAR; indicated by white filled arrowheads) at high density in the subiculum of 4- and 6-month Rag-5xFAD mice. These were often directly adjacent to or very near areas of Aβ accumulation (blue; indicated by white asterisk). Neurons of CA1 also typically exhibited faint to moderate uPAR-ir (white arrows). Young (2-month) and non-5xFAD mice had few ‘activated/ameboid’ Iba1-ir microglia; rather, ‘ramified’ Iba1-ir microglia were evenly distributed throughout these tissues (unfilled, white outline arrowhead). Scale bars: 100 μm (lower mag panels, first column), 50 μm (higher mag panels).

Triple immunofluorescence labeling confirmed uPAR expression primarily in Iba-ir microglial cells, with variable presence in NeuN-ir neurons (Fig. 5; Supp Fig. 1), and minimal expression of uPAR in GFAP-ir astrocytes (Supp Fig. 2). In older Rag-5xFAD (4 or 6 months) and 5xFAD (6 months) animals, uPAR+/IbaI + microglia were very frequently observed and often appeared activated, with large, ameboid cell bodies and few, shorter processes (Fig. 5 filled arrowheads; Supp Fig. 1 filled arrowheads). While uPAR−/Iba1 + resting microglia were fairly evenly distributed throughout the brain (Fig. 5 and Supp Fig. 2 unfilled, white outline arrowheads), labeling with the fluorescent amyloid-beta (Aβ) probe methoxy-X04 revealed that uPAR+/IbaI + microglia were often clustered around areas of Aβ accumulation (Fig. 5 white asterisk; Supp Fig. 2 white asterisk) in the 5xFAD and Rag-5xFAD mice. Microglia with intense uPAR-ir were particularly evident in the subiculum, consistent with brightfield immunolabeling and quantification.

### 3.3. Transcriptional profiling revealed microglial dysfunction and senescence upregulation

**Fig. 6.**
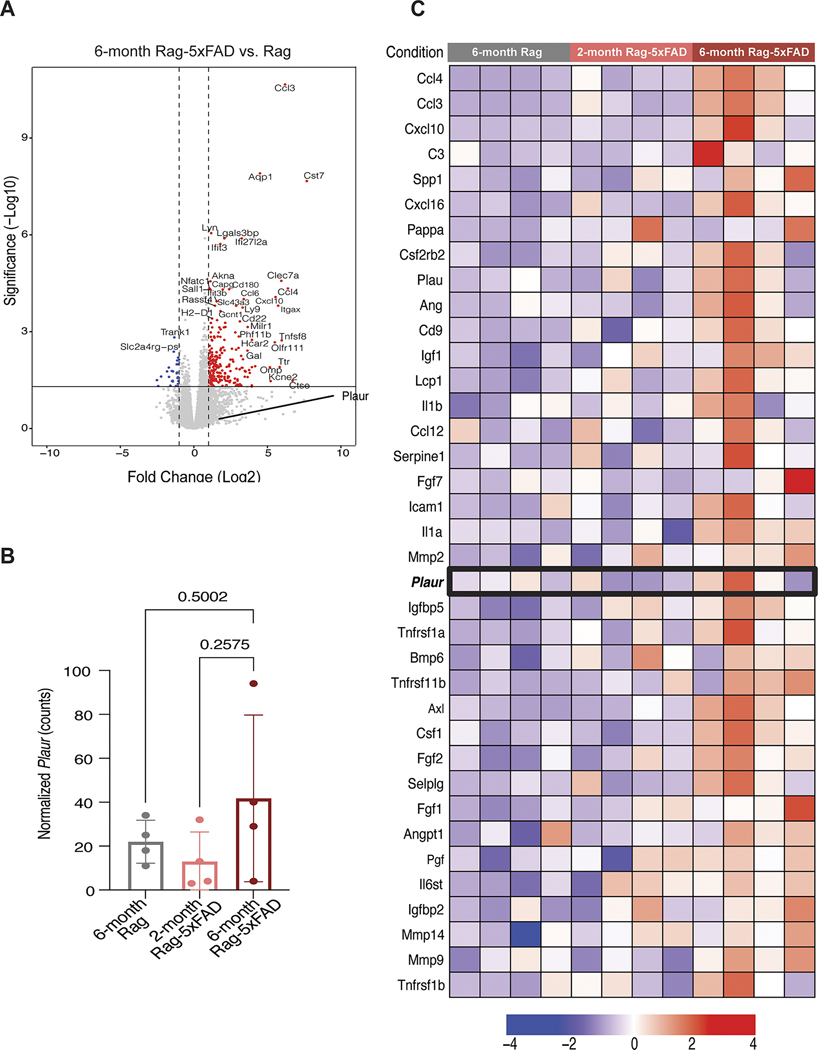
Transcriptomic analysis revealed upregulated senescence-associated gene expression in older Rag-5xFAD mice. (A) Volcano plot of all genes in 6-month Rag-5xFAD compared to 6-month Rag animal controls. Significant up-regulated genes (red; higher in Rag-5xFAD) based on Log2 Fold Change > 1 or < −1; non-significant-genes (grey). The *Plaur* gene is indicated by solid line. (B) Normalized gene count for *Plaur* using DESeq2 package in R across all animal conditions. *Plaur* expression matches previously established aged mouse tissue analysis trends (Amor et al., 2024). Statistical analysis using one-way ANOVA (n = 4 per experimental group). (C) Heatmap visualizing the expression of the leading edge expression of senescent-associated genes from pairwise GSEA analysis (Saul et al., 2022). Genes are ranked top-to-bottom based on their differential expression using the signal-to-noise metric between 6- and 2-month Rag-5xFAD and 6-month Rag mice. Maximum relative expression (red); minimum relative expression (blue).

Differential gene expression analyses were performed in brain tissues, between 6-month old Rag-5xFAD vs. 6-month old Rag mice (Fig. 6A), and 2- vs. 6-month old Rag-5xFAD mice (Supp Fig. 4A). In the 6-month old Rag-5xFAD vs. Rag comparison, 578 genes were differentially regulated, including 484 upregulated (red) and 94 downregulated (blue) genes in the older, Rag-5xFAD animals (Fig. 6A). The majority of upregulated transcripts represented glial markers linked to AD pathogenesis, along with chemokine and cytokine signaling mediators associated with neuroinflammation and cognitive decline (Fig. 6A, Supp Fig. 4A).

Gene ontology (GO) enrichment of 6-month old Rag-5xFAD vs. Rag samples revealed pathways related to glial cell activation and innate immune signaling. Prominent GO terms included innate immune response, pattern recognition receptor signaling, phagocytosis, and cell surface receptor signaling (Supp Fig. 3B). Differentially expressed genes associated with disease-associated microglia (DAM), phagocytosis, and AD (Supp Figs. 3 and 4) were *Trem2, Apoe, Axl, Cst7, Ctsl, Csf1, Clec7a, Itgax* (Keren-Shaul et al., 2017; Yin et al., 2023). Few genes were differentially expressed between 2 and vs. 6-month-old Rag-5xFAD mice (Supp Fig. 4A), suggesting that major transcriptional shifts occur earlier in disease development in immunodeficient AD mice. Consistent with GO findings, DAM- and phagocytosis-related genes were upregulated in 6-month old Rag-5xFAD mice relative 2 month Rag-5xFAD mice (Supp Fig. 4C-D).

Next, we performed gene set enrichment analysis (GSEA) and plotted the ranked genes based on the leading edge of differential expression for aging senescent-associated genes from the mouse SenMayo gene set with a *p*-value < 0.05 (Saul et al., 2022) (Fig. 6C). We observed high positive correlation with senescent-associated genes in our overall GSEA in 6-month Rag-5xFAD mice compared to 6-month Rag and 2-month Rag-5xFAD controls (Supp Fig. 5). Note that *Plaur* transcripts were detected at all time points and upregulated in 6-month Rag-5xFAD mice (Fig. 6B; Fig. 6C black box) although it was not identified as differentially expressed between the 6-month old Rag-5xFAD vs. Rag and or the 2- vs. 6-month old Rag-5xFAD groups (Supp Tables 3 and 4).

## 4. Discussion

These results demonstrated that uPAR expression increased with age in the brains of 5xFAD mice, with a rapid increase observed in Rag-5xFAD mice, aligning with previous reports of age-dependent amyloid accumulation in these models (Marino et al., 2023; Marsh et al., 2016). uPAR-ir was predominantly detected in plaque-associated microglia across immune backgrounds. Lastly, older Rag-5xFAD mice exhibited a DAM-like transcriptional profile with elevated *Plaur*, linking uPAR to microglial dysfunction. Together, these findings highlight an association between uPAR expression, microglial activation states, and disease progression in a model of AD pathology.

In our study, plaque-associated microglia strongly expressed uPAR-ir as early as 4 months of age, and uPAR protein expression levels were significantly increased by 6 months in both 5xFAD and Rag-5xFAD mice. In AD, several cell types, including microglia, astrocytes, oligodendrocytes, and oligodendrocyte precursor cells have been reported to adopt a senescent-like signature (Guerrero et al., 2021; Melo Dos Santos et al., 2024). Previous studies have described expression of uPAR by microglia as characteristic of senescent and/or DAM, possibly reflecting activation or adaptive responses associated with these states (Liu and Chen, 2024). The upregulation of senescent- and DAM-associated genes in our older Rag-5xFAD mice supports this concept, suggesting microglia undergo transcriptional reprogramming toward dysfunctional or altered activation states (Hu et al., 2021; Rim et al., 2024). Notably, the rapid and intense accumulation of uPAR-ir microglia in the subiculum in 5xFAD and Rag-5xFAD animals is in line with previous reports of early Aβ accumulation in this region in 5xFAD mice (Forner et al., 2021), which is also among the earliest regions impacted in clinical AD (Carlesimo et al., 2015).

The increased uPAR protein expression in our female mice relative to male is consistent with reports of increased Aβ accumulation and inflammation in female 5xFAD mice (Poon et al., 2023; Sil et al., 2022) and parallels the higher prevalence of clinical AD in women relative to men (Lopez-Lee et al., 2024). It should be noted that, due to difficulties in sample collection, the sample number of male and female animals in this dataset was not always evenly matched (Supp Table 1), which may have impacted the findings.

Our detection of uPAR-ir in neurons contributes to an area of active debate and research. Previous studies on uPAR expression in mature, adult neurons have produced differing results (O. Cunningham et al., 2009; Del Bigio et al., 1999; Diaz et al., 2019; Lahtinen et al., 2009; Liu et al., 2010; Wu et al., 2014). In the animals evaluated here, low level uPAR-ir (compared to intense uPAR-ir in activated microglia) was consistently observed in limited neuronal populations across all ages and genotypes, particularly CA1 hippocampal neurons, and occasionally in lower cortical level M1 neurons or other regions. Although a number of previous reports observed mature neuronal uPAR-ir under normal conditions (Del Bigio et al., 1999; Liu et al., 2010), others have described more limited neuronal uPAR expression during neurodevelopment and/or following neuronal damage (Del Bigio et al., 1999; Liu et al., 2010; Matsui et al., 2022; Merino et al., 2017). Future studies should aim to quantify uPAR-ir across neuronal populations in this model and to define whether neuronal uPAR contributes to neuroprotective signaling or synaptic dysfunction and neurodegeneration.

It should be noted that the mouse strains used in this study were maintained on two different C57BL/6 substrain backgrounds. WT and 5xFAD mice were kept on a C57BL/6J background, whereas Rag2/Il2rg^−/−^ and Rag-5xFAD mice were on a C57BL/6NTac background. Although these substrains are both derived from the C57BL/6 lineage, they are maintained independently and exhibit some known genetic differences. For example, C57BL/6J mice carry a spontaneous deletion of exons 7–11 in the nicotinamide nucleotide transhydrogenase (*Nnt*) gene, which is not present in C57BL/6N-derived substrains, including C57BL/6NTac (Mekada et al., 2009). This deletion in C57BL/6J mice has been associated with alterations in glucose metabolism in some experimental contexts and mild impairment in glucose tolerance compared to C57BL/6N substrains (Mekada et al., 2009; Nemoto et al., 2022). Additionally, some limited differences in neutrophil recruitment (Ulland et al., 2016; Yamada et al., 2025) and certain behavioral endpoints related to aging (Yamada et al., 2025) have been described between C57BL/6J and C57BL/6NTac mice. To our knowledge, no reports directly comparing uPAR expression or amyloid pathology between these substrains are currently available, and, thus, potential background effects should be considered when interpreting comparisons across immune-competent and immunodeficient cohorts. Moreover, data on Rag-5xFAD mice remain limited (Faridar et al., 2022; Marsh et al., 2017, 2016), with gaps in knowledge regarding these mice survival, lifespan, and cognitive function that warrant future investigation.

Bulk RNA-seq analysis revealed upregulation and differential expression of glial activation markers, innate immune pathways, and senescence- and DAM-associated genes in 6-month Rag-5xFAD mice compared to age-matched Rag mice controls (Grubman et al., 2021; Keren-Shaul et al., 2017). These transcriptional changes are consistent with the detected prominent accumulation of increased uPAR-ir microglia observed by imunostaining. We did not observe a significant difference in differential gene expression between 2- and 6-month Rag-5xFAD mice, suggesting that transcription of senescence- and DAM-related factors may start early and, over time, contribute to pathological progression in this AD model (Rim et al., 2024). Although *Plaur* did not meet statistical thresholds for differential expression, its transcript levels were consistently detected across all investigated groups and increased in 6-month old Rag-5xFAD mice, aligning with the observed trends in uPAR expression in the animals evaluated. These modest changes in *Plaur* transcript abundance are expected, as mRNA abundance has been shown in other research to not correlate linearly with surface uPAR protein levels, indicating that uPAR is likely subject to post-transcriptional regulatory mechanisms (Amor et al., 2024; Stewart and Sayers, 2009).

Together, these findings suggest that uPAR marks microglial populations undergoing transcriptional reprogramming toward DAM-like and inflammatory states; however, whether this reflects a pathogenic or compensatory response to ongoing neurodegeneration and immune activation remains unclear. Prior work has shown that murine *Plaur* can function as an activity-dependent early response gene to neuronal activity and cognitive demand (Klimovich et al., 2025; Shmakova et al., 2020). Moreover, *Plaur*-deficient mice exhibit increased anxiety-like behavior and impaired social interactions, raising the possibility that altered uPAR expression may, in part, represent a compensatory response to heightened DAM activation (Levitt, 2005; Powell et al., 2003). Evidence of *PLAUR* involvement in human neurodevelopmental or neurodegenerative diseases is more limited (Bolkvadze et al., 2016), though the *PLAUR* promoter variant rs344781 has been linked to autism spectrum disorder (Campbell et al., 2008). Future studies leveraging single-cell approaches, rather than bulk RNA-seq, will be critical to resolve cell-type-specific transcriptional changes and more precisely define the role of uPAR/*Plaur*-expressing microglia in disease progression.

Lastly, our results suggest that modulation or selective targeting of uPAR^+^ microglial populations may represent a potential therapeutic strategy for AD. Further evaluation of the context-dependent roles of uPAR, is needed to ascertain whether reducing or preserving these cells would be beneficial for restoring homeostatic functions or limiting neuroinflammation (Lau et al., 2023). The Rag-5xFAD system will be especially valuable (Bader et al., 2023; Forner et al., 2021) for testing how manipulation of uPAR^+^ or DAM-like populations influences neuroinflammatory states and disease progression, and for determining whether targeting the uPA/uPAR axis has therapeutic benefit in AD.

## 5. Conclusion

This study identified uPAR as a marker of glial populations undergoing transcriptional and functional changes associated with DAM-like and senescent-related states in AD-pathology mouse models and correlates expression with age, sex, and immune status. The enrichment of uPAR in plaque-associated microglia suggests that uPAR expression marks activated or reprogrammed microglial populations. While neuronal uPAR expression was limited, further investigation of its cell-specific role will be critical. Overall, these findings provide mechanistic insight into the regulation of uPAR associated with AD pathology. The results lay a foundation for future studies on whether uPAR upregulation reflects pathogenic, compensatory, or context-dependent responses during AD progression, and whether selectively targeting uPAR-expressing glial cells can reduce neuroinflammation and improve functional outcomes in neurodegeneration.

## Supplementary Material

MMC3

MMC10

MMC11

MMC9

MMC8

MMC7

MMC6

MMC2

MMC5

MMC1

MMC4

## Data Availability

Data will be made available on request.

## Appendix A. Supplementary data

Supplementary data to this article can be found online at https://doi.org/10.1016/j.brainres.2026.150364.
