# Supplementary figures and images for: uPAR exhibits age- and region-dependent expression in the brains of mice with Alzheimer’s disease-like pathology
Source: Brain Res. Author manuscript; Available in PMC 2026 Jun 15. (PMC13267882; doi:10.1016/j.brainres.2026.150364)

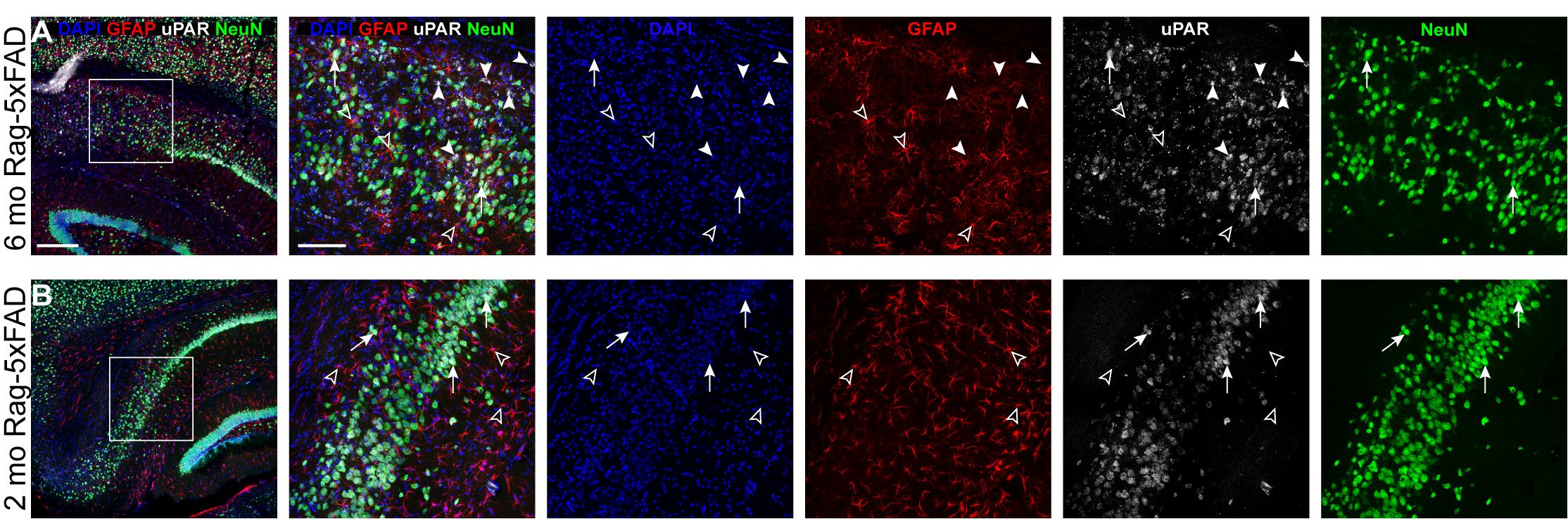

Supplement: MMC3 [file NIHMS2175204-supplement-MMC3.jpg]

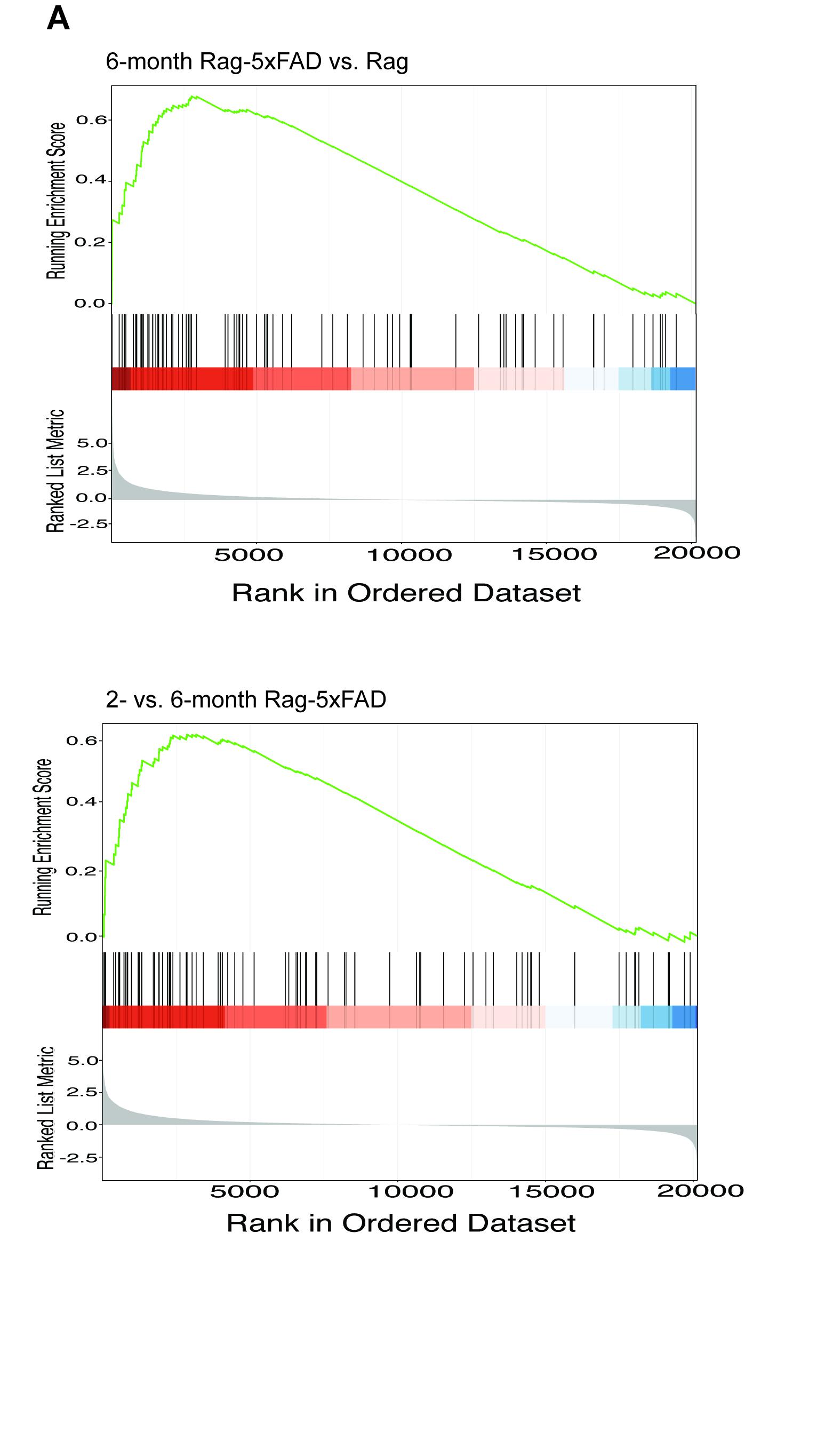

Supplement: MMC6 [file NIHMS2175204-supplement-MMC6.jpg]

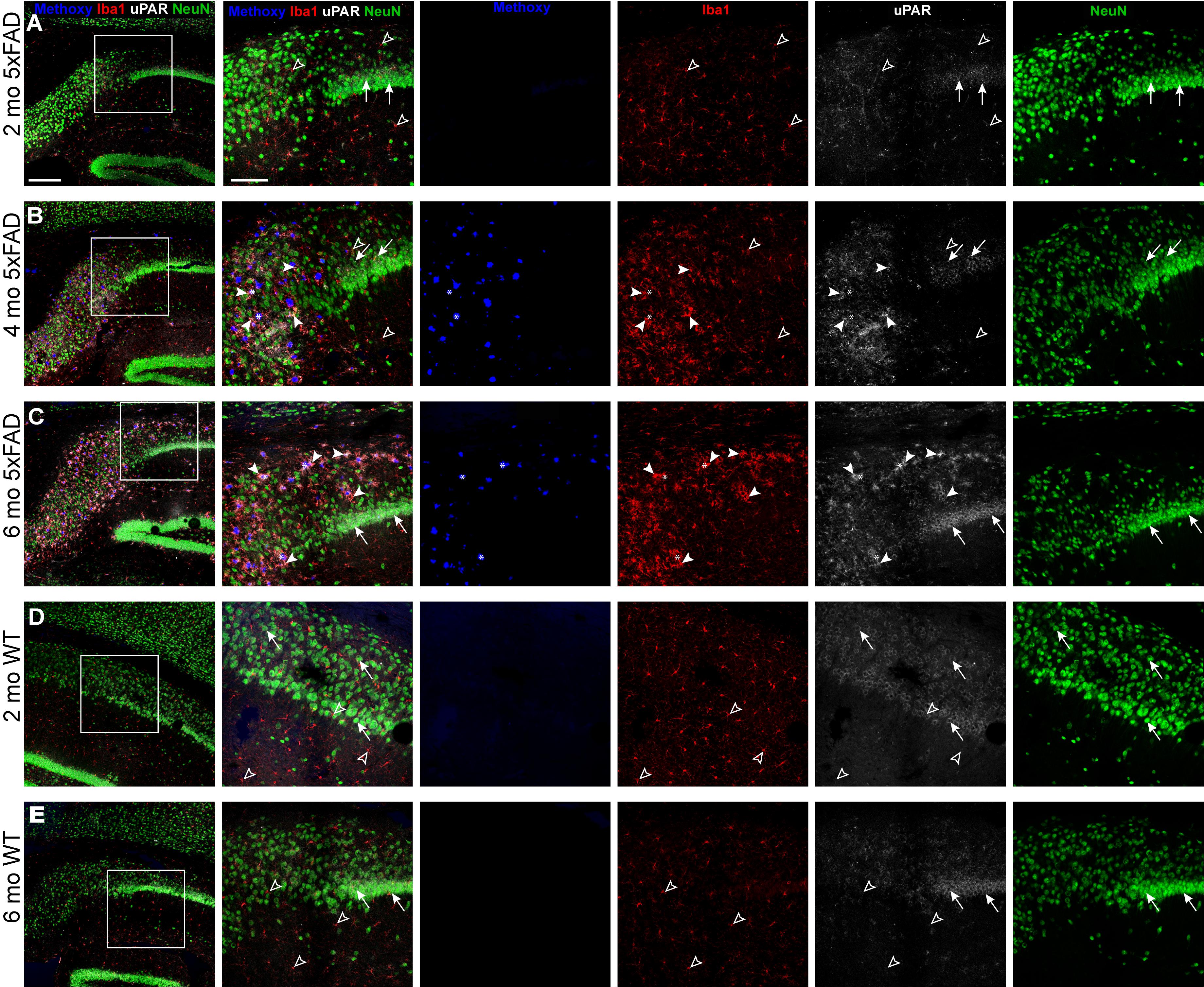

Supplement: MMC2 [file NIHMS2175204-supplement-MMC2.jpg]

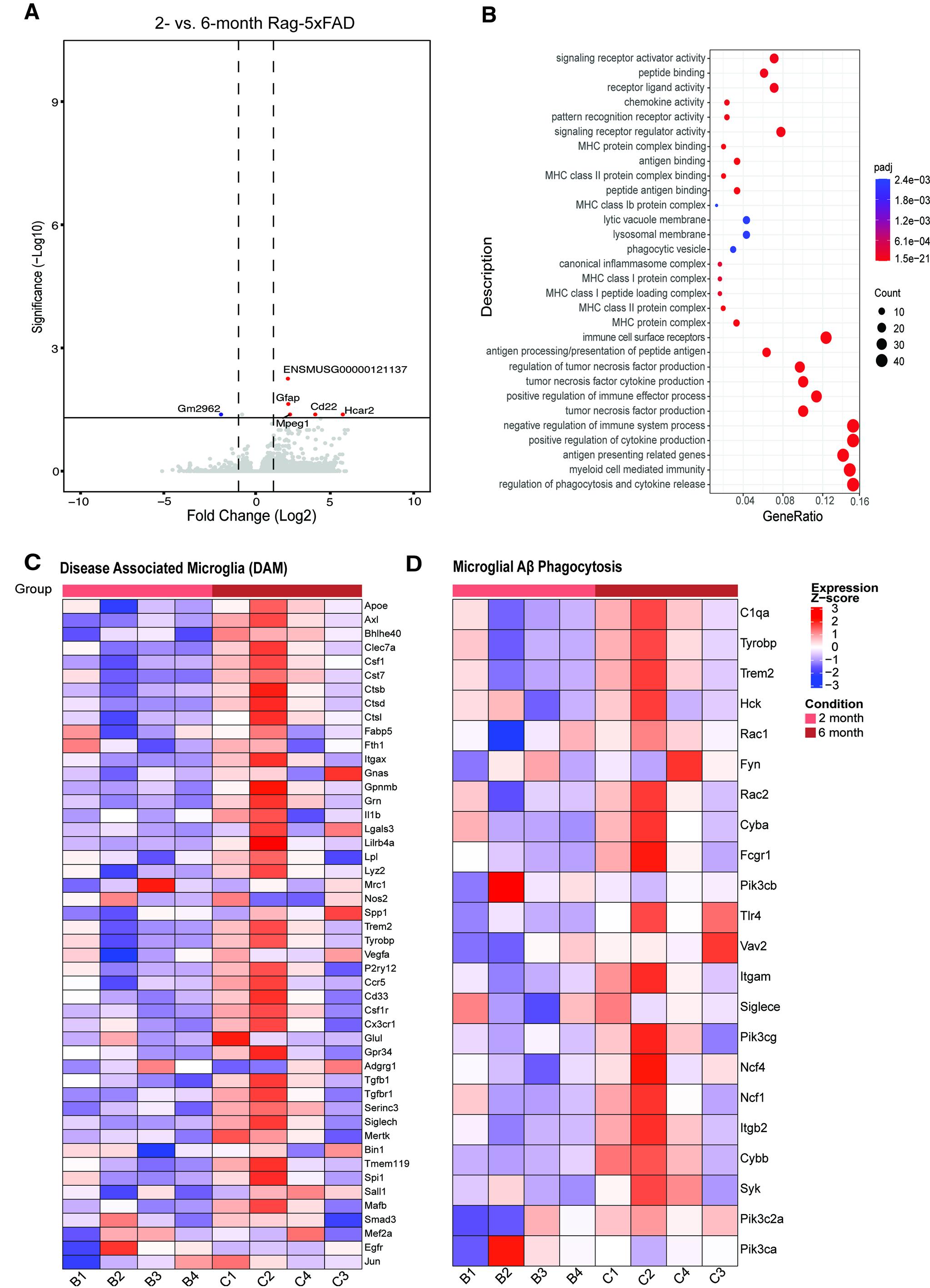

Supplement: MMC5 [file NIHMS2175204-supplement-MMC5.jpg]

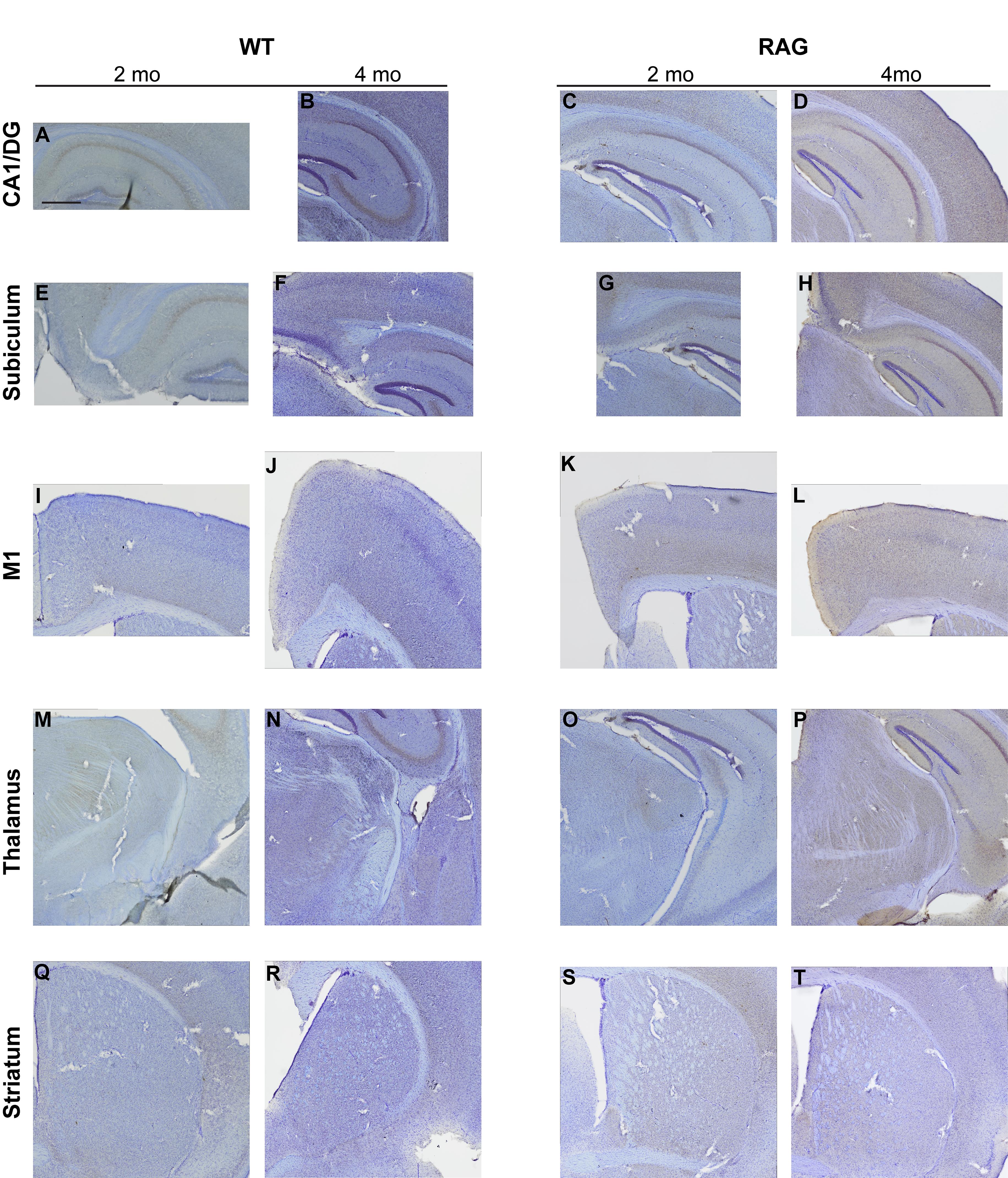

Supplement: MMC1 [file NIHMS2175204-supplement-MMC1.jpg]

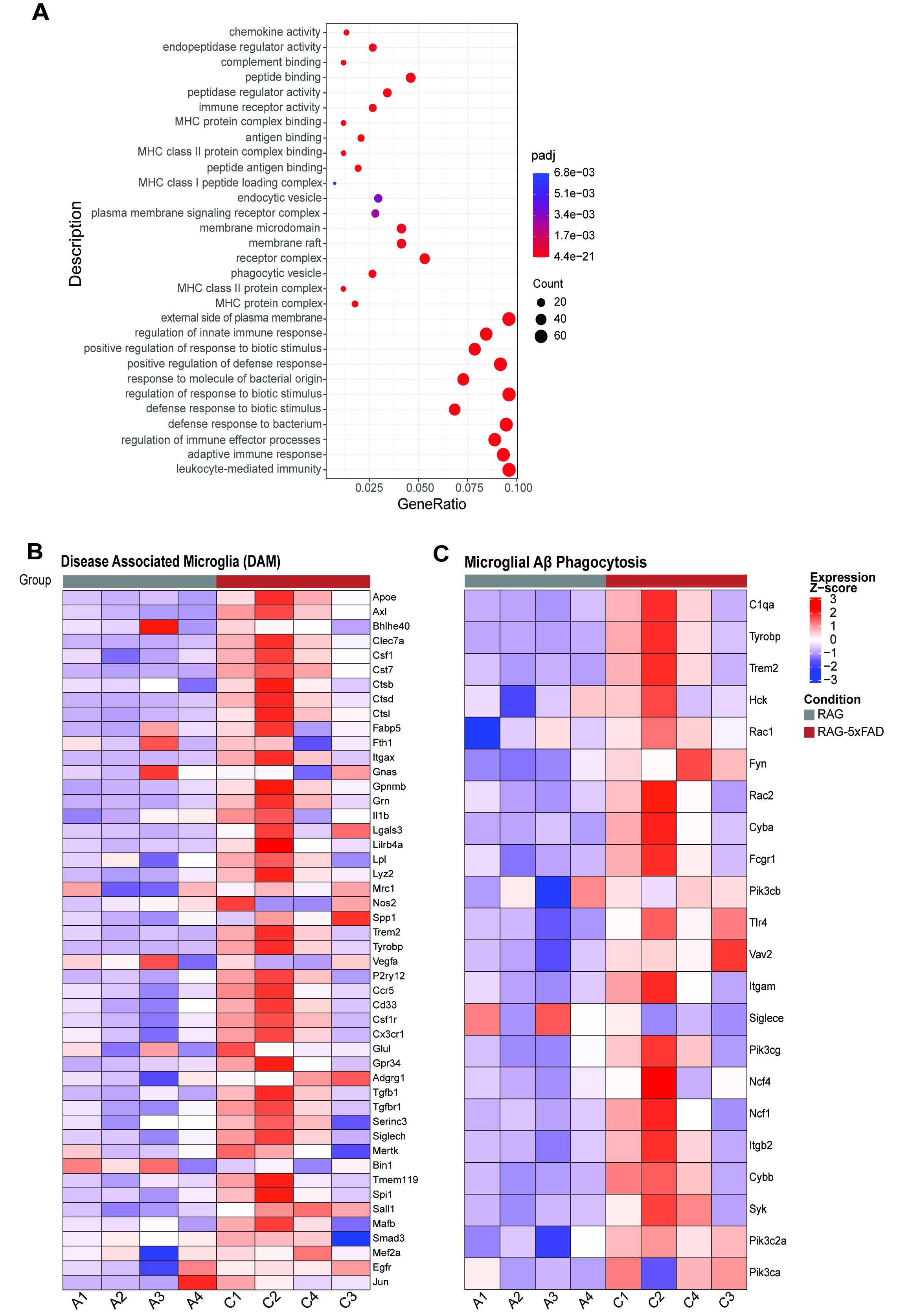

Supplement: MMC4 [file NIHMS2175204-supplement-MMC4.jpg]
