# Supplementary material for: uPAR exhibits age- and region-dependent expression in the brains of mice with Alzheimer’s disease-like pathology
Source: Brain Res. Author manuscript; Available in PMC 2026 Jun 15. (PMC13267882; doi:10.1016/j.brainres.2026.150364)
Supplement: MMC9 [file NIHMS2175204-supplement-MMC9.docx]

**Table 2.** Results of statistical analysis of uPAR-ir percent area above threshold (%AAT) across animal ages, genotypes, and brain regions (ROIs). Results were generated via 4 way (Age*Genotype*Sex*Brain ROI) repeated measures analysis of variance (ANOVA) with sex, genotype, and age as between-subjects factors and Brain ROI as the repeated measures, within-subjects factor. Results of pairwise comparisons are adjusted for multiple comparisons using the Bonferroni correction.

**Table 2.1.** Between and within subject effects

- Within subject effects are Greenhouse-Geisser corrected

| **Effect** | **P value** | **Effect size (partial eta squared)** |
| --- | --- | --- |
| Age | 6.020E-07 | 4.863E-01 |
| Genotype | 2.680E-10 | 6.651E-01 |
| Sex | 2.134E-02 | 1.172E-01 |
| Age*genotype | 2.325E-06 | 5.558E-01 |
| Age*genotype*sex | 3.394E-04 | 4.273E-01 |
| Region | 7.299E-13 | 4.124E-01 |
| Region * age | 5.765E-06 | 2.535E-01 |
| Region * genotype | 1.297E-12 | 4.756E-01 |
| Region*age*genotype | 9.628E-05 | 3.219E-01 |

**Table 2.2.** Post Hoc Tests, genotype differences

- Post hoc tests comparing genotypes without separating ages

| **Comparison** | **P value** |
| --- | --- |
| 5xFAD v WT | 6.643E-05 |
| 5xFAD v Rag | 5.709E-06 |
| Rag v Rag-5xFAD | 7.377E-09 |

**Table 2.3.** Age*Genotype, age differences

| **Genotype** | **Age Comparison (months)** | **P value** |
| --- | --- | --- |
| 5xFAD | 2 v 6 | 9.866E-08 |
| 5xFAD | 4 v 6 | 4.366E-07 |
| Rag-5xFAD | 2 v 4 | 4.329E-05 |
| Rag-5xFAD | 2 v 6 | 6.487E-06 |

**Table 2.4.** Age*Genotype, genotype differences

| **Age (months)** | **Genotype Comparison** | **P value** |
| --- | --- | --- |
| 4 | 5xFAD v Rag-5xFAD | 2.102E-04 |
| 4 | Rag v Rag-5xFAD | 1.254E-05 |
| 4 | WT v Rag-5xFAD | 6.222E-06 |
| 6 | WT v 5xFAD | 3.323E-07 |
| 6 | WT v Rag-5xFAD | 9.670E-06 |
| 6 | 5xFAD v Rag | 4.096E-09 |
| 6 | Rag v Rag-5xFAD | 1.899E-07 |

**Table 2.5.** Age*Genotype*Sex, age differences

| **Genotype** | **Sex** | **Age Comparison (months)** | **P value** |
| --- | --- | --- | --- |
| 5xFAD | F | 2 v 6 | 4.538E-10 |
| 5xFAD | F | 4 v 6 | 8.933E-09 |
| Rag5xFAD | F | 2 v 4 | 2.984E-05 |
| Rag5xFAD | F | 2 v 6 | 6.692E-03 |
| Rag5xFAD | M | 2 v 6 | 1.935E-04 |
| Rag5xFAD | M | 4 v 6 | 2.842E-02 |

**Table 2.6.** Age*Genotype*Sex, genotype differences

| **Age (months)** | **Sex** | **Genotype comparison** | **P value** |
| --- | --- | --- | --- |
| 4 | F | 5xFAD v Rag-5xFAD | 1.300E-05 |
| 4 | F | Rag v Rag5xFAD | 1.056E-05 |
| 4 | F | WT v Rag5xFAD | 5.905E-07 |
| 6 | F | 5xFAD v Rag | 8.707E-10 |
| 6 | F | 5xFAD v Rag5xFAD | 3.368E-03 |
| 6 | F | 5xFAD v WT | 5.907E-09 |
| 6 | F | Rag v Rag5xFAD | 1.968E-04 |
| 6 | F | Rag5xFAD v WT | 1.261E-03 |
| 6 | M | 5xFAD v Rag | 4.683E-02 |
| 6 | M | Rag v Rag5xFAD | 8.948E-05 |
| 6 | M | WT vs Rag5xFAD | 2.214E-03 |

**Table 2.7.** Sex Differences

- age*sex or genotype*sex – averaged over regions

| **Age (months)** | **Genotype** | **M v F p value** |
| --- | --- | --- |
| 4 | Rag-5xFAD | 1.465E-04 |
| 6 | 5xFAD | 7.905E-07 |

**Table 2.8.** Age*Genotype*Region, sex differences

| **Genotype** | **Region** | **Age Comparison (months)** | **P value** |
| --- | --- | --- | --- |
| 5xFAD | CA1 | 2 v 6 | 1.190E-03 |
| 5xFAD | CA1 | 4 v 6 | 6.772E-04 |
| 5xFAD | DG | 2 v 6 | 9.673E-03 |
| 5xFAD | DG | 4 v 6 | 3.494E-04 |
| 5xFAD | Subiculum | 2 v 6 | 1.237E-06 |
| 5xFAD | Subiculum | 4 v 6 | 1.906E-04 |
| 5xFAD | M1 | 2 v 6 | 3.547E-05 |
| 5xFAD | M1 | 4 v 6 | 3.369E-04 |
| 5xFAD | Thalamus | 2 v 6 | 5.717E-03 |
| 5xFAD | Thalamus | 4 v 6 | 2.296E-02 |
| Rag-5xFAD | Subiculum | 2 v 4 | 1.575E-05 |
| Rag-5xFAD | Subiculum | 2 v 6 | 5.163E-08 |
| Rag-5xFAD | M1 | 2 v 6 | 3.477E-03 |
| Rag-5xFAD | Thalamus | 2 v 4 | 5.620E-04 |

**Table 2.9.** Age*Genotype*Region, genotype differences

| **Age (months)** | **Region** | **Genotype Comparison** | **P value** |
| --- | --- | --- | --- |
| 4 | Subiculum | 5xFAD v Rag-5xFAD | 4.813E-04 |
| 4 | Subiculum | Rag v Rag-5xFAD | 8.264E-06 |
| 4 | Subiculum | WT v Rag-5xFAD | 8.480E-07 |
| 4 | M1 | 5xFAD v Rag-5xFAD | 4.623E-02 |
| 4 | M1 | Rag v Rag-5xFAD | 7.394E-03 |
| 4 | M1 | WT v Rag-5xFAD | 2.695E-03 |
| 4 | Striatum | 5xFAD v Rag-5xFAD | 3.345E-02 |
| 4 | Striatum | WT v Rag-5xFAD | 2.765E-02 |
| 4 | Thalamus | 5xFAD v Rag-5xFAD | 3.959E-03 |
| 4 | Thalamus | Rag v Rag-5xFAD | 2.104E-03 |
| 4 | Thalamus | WT v Rag-5xFAD | 4.401E-04 |
| 6 | CA1 | 5xFAD v Rag | 2.210E-05 |
| 6 | CA1 | 5xFAD v WT | 1.017E-03 |
| 6 | DG | 5xFAD v Rag | 3.336E-04 |
| 6 | Subiculum | 5xFAD v Rag | 6.878E-07 |
| 6 | Subiculum | 5xFAD v WT | 2.443E-06 |
| 6 | Subiculum | Rag v Rag-5xFAD | 3.642E-09 |
| 6 | Subiculum | Rag-5xFAD v WT | 1.454E-08 |
| 6 | M1 | 5xFAD v Rag | 2.180E-05 |
| 6 | M1 | 5xFAD v WT | 9.640E-05 |
| 6 | M1 | Rag v Rag-5xFAD | 7.149E-05 |
| 6 | M1 | Rag-5xFAD v WT | 2.570E-04 |
| 6 | Thalamus | 5xFAD v Rag | 6.553E-03 |
| 6 | Thalamus | 5xFAD v WT | 1.143E-02 |
| 6 | Thalamus | Rag v Rag-5xFAD | 7.501E-03 |
| 6 | Thalamus | Rag-5xFAD v WT | 1.223E-02 |

**Table 2.10.** Age*Genotype*Region, region differences

| **Age (months)** | **Genotype** | **Region Comparison** | **P value** |
| --- | --- | --- | --- |
| 4 | Rag-5xFAD | CA1 v Subiculum | 3.268E-06 |
| 4 | Rag-5xFAD | DG v Subiculum | 3.886E-04 |
| 4 | Rag-5xFAD | Subiculum v M1 | 8.026E-08 |
| 4 | Rag-5xFAD | Subiculum v Striatum | 1.523E-08 |
| 4 | Rag-5xFAD | Subiculum v Thalamus | 1.012E-07 |
| 4 | Rag-5xFAD | M1 v Striatum | 7.374E-03 |
| 6 | 5xFAD | CA1 v Subiculum | 1.773E-03 |
| 6 | 5xFAD | CA1 v Striatum | 3.966E-08 |
| 6 | 5xFAD | CA1 v Thalamus | 3.061E-03 |
| 6 | 5xFAD | DG v M1 | 1.117E-02 |
| 6 | 5xFAD | DG v Striatum | 2.483E-07 |
| 6 | 5xFAD | DG v Thalamus | 4.251E-03 |
| 6 | 5xFAD | Subiculum v M1 | 1.012E-06 |
| 6 | 5xFAD | Subiculum v Striatum | 3.097E-09 |
| 6 | 5xFAD | Subiculum v Thalamus | 1.414E-08 |
| 6 | 5xFAD | M1 v Striatum | 2.946E-09 |
| 6 | Rag-5xFAD | CA1 v Subiculum | 4.398E-09 |
| 6 | Rag-5xFAD | DG v Subiculum | 8.677E-07 |
| 6 | Rag-5xFAD | Subiculum v M1 | 8.282E-10 |
| 6 | Rag-5xFAD | Subiculum v Striatum | 1.729E-11 |
| 6 | Rag-5xFAD | Subiculum v Thalamus | 3.177E-11 |
| 6 | Rag-5xFAD | M1 v Striatum | 2.677E-07 |

**Table 2.11.** Age*Genotype*Sex*Region, age differences

| **Genotype** | **Sex** | **Region** | **Age Comparison (months)** | **P value** |
| --- | --- | --- | --- | --- |
| 5xFAD | F | CA1 | 2 v 6 | 6.268E-06 |
| 5xFAD | F | CA1 | 4 v 6 | 4.468E-05 |
| 5xFAD | F | DG | 2 v 6 | 6.459E-05 |
| 5xFAD | F | DG | 4 v 6 | 6.530E-05 |
| 5xFAD | F | Subiculum | 2 v 6 | 4.709E-07 |
| 5xFAD | F | Subiculum | 4 v 6 | 1.718E-05 |
| 5xFAD | F | M1 | 2 v 6 | 1.409E-06 |
| 5xFAD | F | M1 | 4 v 6 | 1.460E-05 |
| 5xFAD | F | Striatum | 2 v 6 | 3.296E-02 |
| 5xFAD | F | Striatum | 4 v 6 | 4.136E-02 |
| 5xFAD | F | Thalamus | 2 v 6 | 3.893E-04 |
| 5xFAD | F | Thalamus | 4 v 6 | 2.677E-03 |
| Rag-5xFAD | F | Subiculum | 2 v 4 | 3.826E-05 |
| Rag-5xFAD | F | Subiculum | 2 v 6 | 1.952E-05 |
| Rag-5xFAD | F | Striatum | 2 v 4 | 8.291E-03 |
| Rag-5xFAD | F | Striatum | 4 v 6 | 4.276E-03 |
| Rag-5xFAD | F | Thalamus | 2 v 4 | 8.414E-05 |
| Rag-5xFAD | M | Subiculum | 2 v 6 | 8.544E-05 |
| Rag-5xFAD | M | Subiculum | 4 v 6 | 4.732E-02 |
| Rag-5xFAD | M | M1 | 2 v 6 | 2.396E-04 |
| Rag-5xFAD | M | M1 | 4 v 6 | 4.124E-03 |

**Table 2.12.** Age*Genotype*Sex*Region, genotype differences

| **Age (months)** | **Sex** | **Region** | **Genotype Comparison** | **P value** |
| --- | --- | --- | --- | --- |
| 4 | F | DG | WT v Rag-5xFAD | 6.963E-02 |
| 4 | F | Subiculum | 5xFAD v Rag-5xFAD | 3.324E-05 |
| 4 | F | Subiculum | Rag v Rag-5xFAD | 7.247E-06 |
| 4 | F | Subiculum | WT v Rag-5xFAD | 7.826E-07 |
| 4 | F | M1 | 5xFAD v Rag-5xFAD | 7.896E-03 |
| 4 | F | M1 | Rag v Rag-5xFAD | 2.617E-03 |
| 4 | F | M1 | WT v Rag-5xFAD | 7.720E-04 |
| 4 | F | Striatum | 5xFAD v Rag-5xFAD | 2.253E-03 |
| 4 | F | Striatum | Rag v Rag-5xFAD | 6.344E-03 |
| 4 | F | Striatum | WT v Rag-5xFAD | 1.694E-03 |
| 4 | F | Thalamus | 5xFAD v Rag-5xFAD | 4.030E-04 |
| 4 | F | Thalamus | Rag v Rag-5xFAD | 2.863E-04 |
| 4 | F | Thalamus | WT v Rag-5xFAD | 5.094E-05 |
| 6 | F | CA1 | 5xFAD v Rag | 1.424E-05 |
| 6 | F | CA1 | 5xfAD v Rag-5xFAD | 3.919E-04 |
| 6 | F | CA1 | 5xFAD v WT | 2.949E-04 |
| 6 | F | DG | 5xFAD v RAG | 1.259E-04 |
| 6 | F | DG | 5xfAD v Rag-5xFAD | 2.648E-03 |
| 6 | F | DG | 5xFAD v WT | 2.013E-03 |
| 6 | F | Subiculum | 5xFAD v RAG | 8.506E-07 |
| 6 | F | Subiculum | 5xFAD v WT | 8.909E-07 |
| 6 | F | Subiculum | Rag v Rag-5xFAD | 4.132E-07 |
| 6 | F | Subiculum | Rag-5xFAD v WT | 4.328E-07 |
| 6 | F | M1 | 5xFAD v Rag | 2.226E-06 |
| 6 | F | M1 | 5xFAD v Rag-5xFAD | 4.371E-03 |
| 6 | F | M1 | 5xFAD v WT | 1.978E-06 |
| 6 | F | Striatum | 5xFAD v Rag | 7.296E-02 |
| 6 | F | Striatum | 5xFAD v WT | 7.868E-02 |
| 6 | F | Thalamus | 5xFAD v Rag | 8.038E-04 |
| 6 | F | Thalamus | 5xFAD v WT | 7.602E-04 |
| 6 | F | Thalamus | Rag v Rag-5xFAD | 4.751E-02 |
| 6 | F | Thalamus | Rag-5xFAD v WT | 4.535E-02 |
| 6 | M | Subiculum | Rag v Rag-5xFAD | 1.095E-04 |
| 6 | M | Subiculum | Rag-5xFAD v WT | 4.321E-04 |
| 6 | M | M1 | 5xFAD v Rag-5xFAD | 1.646E-02 |
| 6 | M | M1 | Rag v Rag-5xFAD | 2.752E-04 |
| 6 | M | M1 | Rag-5xFAD v WT | 1.835E-03 |

**Table 2.13.** Age*Genotype*Sex*Region, sex differences

| **Age (months)** | **Genotype** | **Region** | **M v F p value** |
| --- | --- | --- | --- |
| 4 | Rag-5xFAD | DG | 2.848E-02 |
| 4 | Rag-5xFAD | Subiculum | 5.581E-04 |
| 4 | Rag-5xFAD | M1 | 4.420E-03 |
| 4 | Rag-5xFAD | Striatum | 6.988E-04 |
| 4 | Rag-5xFAD | Thalamus | 3.234E-04 |
| 6 | 5xFAD | CA1 | 4.511E-04 |
| 6 | 5xFAD | DG | 1.097E-03 |
| 6 | 5xFAD | Subiculum | 8.073E-04 |
| 6 | 5xFAD | M1 | 4.824E-05 |
| 6 | 5xFAD | Striatum | 1.838E-02 |
| 6 | 5xFAD | Thalamus | 1.238E-03 |
| 6 | Rag-5xFAD | M1 | 2.118E-02 |
