# Supplementary material for: uPAR exhibits age- and region-dependent expression in the brains of mice with Alzheimer’s disease-like pathology
Source: Brain Res. Author manuscript; Available in PMC 2026 Jun 15. (PMC13267882; doi:10.1016/j.brainres.2026.150364)
Supplement: MMC8 [file NIHMS2175204-supplement-MMC8.docx]

**Supplemental Table 1.**

**1a: Subjects included in immunohistochemical (IHC) evaluation of uPAR expression.**

| Genotype | Age (months) | n (#male, #female) |
| --- | --- | --- |
| WT | 2 | 6 (3, 3) |
|  | 4 | 6 (3, 3) |
|  | 6 | 5 (2, 3) |
| 5xFAD | 2 | 5 (2, 3) |
|  | 4 | 6 (3, 3) |
|  | 6 | 6 (3, 3) |
| Rag | 2 | 6 (3, 3) |
|  | 4 | 4 (2, 2) |
|  | 6 | 6 (3, 3) |
| Rag-5xFAD | 2 | 6 (3, 3) |
|  | 4 | 6 (3, 3) |
|  | 6 | 5 (2, 3) |

**1b: Subjects included in RNA-seq.**

| Genotype | Age (months) | n (all female) |
| --- | --- | --- |
| Rag | 2 | 2 |
|  | 6 | 2 |
| Rag-5xFAD | 2 | 2 |
|  | 6 | 2 |
|  |  |  |
