# Supplementary material for: uPAR exhibits age- and region-dependent expression in the brains of mice with Alzheimer’s disease-like pathology
Source: Brain Res. Author manuscript; Available in PMC 2026 Jun 15. (PMC13267882; doi:10.1016/j.brainres.2026.150364)
Supplement: MMC7 [file NIHMS2175204-supplement-MMC7.docx]

**Supp Figure 1. WT and Rag mice at 2 and 4 months of age minimally expressed uPAR.** Representative photomicrographs showing uPAR-ir (brown) in Nissl (blue) counterstained coronal mouse brain sections from wild type (WT; A, B, E, F, I, J, M, N, Q, R) and Rag (C, D, G, H, K, L, O, P, S, T) mice. Representative drawings of regions of interest (ROIs) used for quantification of uPAR-ir are shown main text figures 1-3. No difference in uPAR expression was observed between WT and Rag mice at any timepoint or between any ages (2, 4, or 6 months) in either the WT or Rag groups; see main text Figure 4 for quantification. Scale bar (A): 200 µm.

**Supp Figure 2. uPAR-immunoreactivity (-ir) was typically observed in Iba1-ir microglia in older 5xFAD animals, with faint uPAR-ir variably present in NeuN-ir neurons.** Representative photomicrographs of triple label immunofluorescence for the microglial marker Iba1 (red), uPAR (white), and the neuronal marker NeuN (green) counterstained with the fluorescent amyloid-beta (Aβ) probe methoxy-X04 (blue) in 2- (A), 4- (B), and 6-month (C) 5xFAD mice and in 2- (D) and 6-month (E) wild type (WT) mice. A white box in the first column panel indicates the area of higher magnification shown in panels to the right for each row. Microglia immunoreactive for both Iba1 and uPAR appear pink (red Iba-ir white uPAR-ir; indicated by white filled arrowheads) at high density in the subiculum of older 5xFAD mice. These were often directly adjacent to or very near areas of Aβ accumulation (blue; indicated by white asterisk). Neurons of CA1 also typically exhibited faint to moderate uPAR-ir (white arrows). Young (2-month) and non-5xFAD mice had few ‘activated/ameboid’ Iba1-ir microglia; rather, ‘ramified’ Iba1-ir microglia were evenly distributed throughout these tissues (unfilled, white outline arrowhead). Scale bars: 100 µm (lower mag panels, first column), 50 µm (higher mag panels).

**Supp Figure 3. uPAR-immunoreactivity (-ir) was rarely observed in GFAP-ir astrocytes.** Representative photomicrographs of triple label immunofluorescence for the astrocyte marker GFAP (red), uPAR (white), and the neuronal marker NeuN (green) counterstained with the nuclear marker DAPI (blue) in 2- (A) and 6-month (B) Rag-5xFAD mice. A white box in the first column panel indicates the area of higher magnification shown in panels to the right for each row. Both 2- and 6-month Rag-5xFAD mice displayed evenly distributed GFAP-ir astrocytes, which were typically lacking uPAR-ir (unfilled, white outline arrowhead). In 6-month Rag-5xFAD mice, uPAR-ir surrounding blue nuclei (white filled arrowhead) was observed, which was morphologically consistent with uPAR-ir microglia in Figure 5 and Supplemental Figure 1. Neurons of CA1 also typically exhibited faint to moderate uPAR-ir (white arrows). Scale bars: 100 µm (lower mag panels, first column), 50 µm (higher mag panels).

**Supp Figure 4. Transcriptomic analysis reveals microglial dysfunction and senescence in 6-month Rag-5xFAD mice compared to Rag mice.** (A) GO dot enrichment analysis from molecular function, biological process, or cellular pathways of 6-month Rag-5xFAD mice and 6-month Rag mice control. (B-C) Heatmaps generated by differential gene expression of 6-month Rag-5xFAD and Rag 2-month mice involved in (B) neurodegenerative and disease-associated microglial (DAM) genes, (C) microglial Aβ phagocytosis genes. Coded sample identifiers (A1–A4, C1–C4) are shown along the x-axis; corresponding experimental groups are indicated by the annotation bar.

**Supp Figure 5. Transcriptomic analysis revealed microglial dysfunction and senescence in 6-month compared to 2-month Rag-5xFAD mice.** (A) Volcano plot of all genes in 6- vs. 2-month Rag-5xFAD animals. Significant upregulated genes (red) based on Log2 Fold Change > 1; and significant downregulated genes (blue) based on Log2 Fold Change < -1; non-significant (grey). Significant genes 0.05< padj called out with max overlap = 15. (B) GO dot enrichment analysis from molecular function, biological process, or cellular pathways of 6-month Rag-5xFAD mice and 2-month Rag-5xFAD mice. (C-D) Heatmaps generated by differential gene expression of 6-month Rag-5xFAD and 2-month Rag-5xFAD mice involved in (C) neurodegenerative and disease associated microglial genes, (D) microglial Aβ phagocytosis genes. Coded sample identifiers (A1–A4, B1–B4) are shown along the x-axis; corresponding experimental groups are indicated by the annotation bar.

**Supplemental Figure 6. Enrichment plots from GSEA analysis.** (A) GSEA analysis showed that senescence-associated genes from the SenMayo data set were differentially enriched in the 6-month Rag-5xFAD expression phenotype compared to 6-month Rag and 2-month Rag-5xFAD controls. Ranking list metric was generated using signal to noise ratio: high positively correlated expression (red); low negatively correlated expression (blue).
